# Modified lipid metabolism and cytosolic phospholipase A2 activation in mesangial cells under pro-inflammatory conditions

**DOI:** 10.1038/s41598-022-10907-4

**Published:** 2022-05-05

**Authors:** Roberto Boi, Kerstin Ebefors, Marcus Henricsson, Jan Borén, Jenny Nyström

**Affiliations:** 1 Institute of Neuroscience and Physiology, Department of Physiology, Sahlgrenska Academy, University of Gothenburg, Box 432, 40530 Gothenburg, Sweden; 2 Institute of Medicine, Department of Molecular and Clinical Medicine, Wallenberg Laboratory, University of Gothenburg, and Sahlgrenska University Hospital, Gothenburg, Sweden

**Keywords:** Lipidomics, Diabetic nephropathy, Glomerulus

## Abstract

Diabetic kidney disease is a consequence of hyperglycemia and other complex events driven by early glomerular hemodynamic changes and a progressive expansion of the mesangium. The molecular mechanisms behind the pathophysiological alterations of the mesangium are yet to be elucidated. This study aimed at investigating whether lipid signaling might be the missing link. Stimulation of human mesangial cells with high glucose primed the inflammasome-driven interleukin 1 beta (IL-1β) secretion, which in turn stimulated platelet-derived growth factor (PDGF-BB) release. Finally, PDGF-BB increased IL-1β secretion synergistically. Both IL-1β and PDGF-BB stimulation triggered the formation of phosphorylated sphingoid bases, as shown by lipidomics, and activated cytosolic phospholipase cPLA2, sphingosine kinase 1, cyclooxygenase 2, and autotaxin. This led to the release of arachidonic acid and lysophosphatidylcholine, activating the secretion of vasodilatory prostaglandins and proliferative lysophosphatidic acids. Blocking cPLA2 release of arachidonic acid reduced mesangial cells proliferation and prostaglandin secretion. Validation was performed in silico using the Nephroseq database and a glomerular transcriptomic database. In conclusion, hyperglycemia primes glomerular inflammatory and proliferative stimuli triggering lipid metabolism modifications in human mesangial cells. The upregulation of cPLA2 was critical in this setting. Its inhibition reduced mesangial secretion of prostaglandins and proliferation, making it a potential therapeutical target.

## Introduction

Diabetic kidney disease (DKD) is a long-term complication of diabetes mellitus and the main cause of chronic kidney disease and end-stage renal disease worldwide. DKD symptoms are often subtle at an early-stage and progress heterogeneously. DKD is characterized by glomerular hypertrophy and sclerosis, tubulointerstitial fibrosis and mesangial expansion with extra cellular matrix accumulation leading to nephron loss and consequently reduced glomerular filtration rate (GFR)^1^.

It is commonly acknowledged that the hyperglycemic diabetic milieu is a prominent risk factor for the onset of DKD in that it causes activation of the renin–angiotensin–aldosterone system and downstream mediators resulting in vasodilation and glomerular hyperfiltration^2^. Glomerular alterations such as mesangial expansion follow. Obesity and the metabolic syndrome are also closely related to diabetes with key features including pro-inflammatory cytokine release, systemic inflammation, and dysregulation of lipid metabolism^3–5^. Glomerular lipid metabolism dysfunction, partly caused by systemic inflammation, may represent a missing link between the early hemodynamic events and expansion of the mesangium, in time leading to nephron loss and reduced GFR.

One of the pro-inflammatory cytokines involved in diabetes is interleukin 1 beta (IL-1β). Its secretion is increased in type 1 diabetes and has a strong connection with the onset of type 2 diabetes^5^, though it is not clear whether as a cause or as a downstream effect^6,7^. An increasing body of evidence suggest that IL-1β is produced after activation of the inflammasome NLRP3 in glomerular cells in DKD^8^. Accordingly, IL-1β mRNA is expressed in kidney biopsies of DKD patients^9^. Inhibition of NLRP3 activation is reported to ameliorate renal injury in diabetic and DKD mouse models^10,11^. NLRP3 overexpression with consequent IL-1β secretion has been observed in human mesangial cells after high glucose stimulus^12^.

Mesangial cells are not only susceptible to inflammatory processes, but also to proliferative stimuli. The expression of PDGF-B (platelet derived growth factor), the main growth factor responsible for mesangial cells proliferation^13^, is indeed upregulated in DKD^14^. Moreover, the percentage of cells expressing mRNA for PDGF-B and its receptors is higher in biopsies from DKD patients with both type 1 and 2 diabetes compared to human healthy kidneys^15^.

The mesangial cell is central in the development of DKD and we hypothesize that human mesangial cells, when exposed to hyperglycemia and through a synergistic activation of inflammatory and proliferative pathways, modify their lipid metabolism. This in turn would lead to increased proliferation and release of hormones able to trigger haemodynamic alterations. Prostaglandins (PGs), for instance, are products of the cyclooxygenase pathway and can cause vasodilation of the renal afferent arteriole increasing GFR (hyperfiltration), a process that prompts DKD^2,16^. The rate limiting enzyme for PG production is the calcium dependent cytosolic phospholipase A2 group IVA (cPLA2). This enzyme is needed for production of arachidonic acid (AA) that COX-2 uses for production of PGs^17^. A recent publication reveals that IL-1β is able to promote proliferation in mouse mesangial cells through the release of calcium^18^. Interestingly, PDGF-BB is also known to stimulate calcium release in cultured rat mesangial cells^19^. Taken together, these findings suggest a link between inflammation and proliferation having cPLA2 activation as common denominator.

In this study, we show that the interplay between inflammatory and proliferative stimuli, primed by exposure to high glucose, modifies mesangial cells lipid metabolism and activate cPLA2 and COX-2. These events trigger secretory as well as proliferative responses and might explain how early signs of DKD such as mesangial expansion and hemodynamic changes are caused by the diabetic environment.

## Methods

### Cell culture

Human mesangial cells (Cells Systems, Kirkland, WA) were cultured in DMEM F12, 1% P/S (Thermo Fisher Scientific, Hampton, NH), 10% FBS (Hyclone, Logan, UT). Before the experiments, the cells were starved overnight in medium containing 0.5% FBS. The cells were treated with D-Glucose, mannitol (Sigma-Aldrich, St. Louis, MO), IL-1β (R&D systems, Minneapolis, MN), PDGF-BB (R&D systems), PGE2 or iloprost (Sigma-Aldrich). PGI2 could not be used for in vitro experiments due to its instability and thus its stable synthetic analouge iloprost was used, adequately adjusting its concentration since it is ten times more powerful than PGI2^20^. A 1 h pre-treatment with 1 µM AACOCF3 (Abcam, Cambridge, UK) was used for the inhibition of cPLA2.

### Western blot

Pierce BCA protein assay kit was used for protein quantification (Thermo Fisher). The cell lysates were stored at – 80 °C until the day of analysis. Protein analysis was carried out using the Bio-Rad Mini Protean TGX Stain-Free gels system. Electrophoretic gels were run and subsequently activated for 45 s using the ChemiDoc Touch system (Bio-Rad Laboratories, Hercules, CA). Proteins were blotted onto a low fluorescence PVDF membrane using the Trans-Blot Turbo Transfer system (Bio-Rad Laboratories). The membranes were incubated with primary antibodies diluted in 20 mM tris-hydroxymethyl aminomethane, 1% tween 20 (TBST, Sigma-Aldrich), 150 mM NaCl (Merck, Burlington, MS) pH 8.0 containing either 5% milk blocking buffer (Bio-Rad) or BSA 5% (Sigma Aldrich) and incubated overnight at 4 °C. A secondary HRP-conjugate anti-rabbit antibody was applied for 1 h at room temperature (Promega, Madison, WI). Visualization of the bands was obtained with SuperSignal West Femto Maximum Sensitivity Substrate (Thermo Fisher) or Clarity Western ECL solutions (Bio-Rad Laboratories). For relative quantifications, the volume of each band was normalized to the total lane volume using the Image Lab software (Bio-Rad Laboratories), according to the protocol from the manufacturer. The following antibodies were used: anti-Phospho-cPLA2 #53044 (Ser505) and anti-total cPLA2 #2831 (Cell Signaling technology, Danvers, MA), used at a 1:500 v/v dilution in TBST 5% BSA; anti-Sphk1 #12071, anti-NLRP3 #15101, anti-IL-1β #12703, anti-COX-2 #4842 (Cell Signaling technology) used at a 1:500 v/v dilution in 5% milk; finally, anti-Cerk #HPA064699 (Sigma Aldrich), used at a 1:1000 v/v dilution in TBST 5% milk.

### Quantitative real-time PCR

mRNA was extracted using the RNeasy kit (Qiagen, Hilden, Germany) following the manufacturer’s indications and quantified with Nanodrop One (Thermo Scientific, Waltham, MA). cDNA was obtained with the RNA to cDNA kit (Thermo Fisher). A QuantStudio 7 Pro Real-Time PCR System and the Design and Analysis 2 software (both from Thermo Fisher) were used for the expression analysis. VIC conjugated GAPDH was used as housekeeping gene, whereas the other genes of interest were FAM conjugated (CERK, CERS2, ENPP2, LPCAT1, LPCAT4, NLRP3, PLA2G4A, PLA2G4B, PLA2G4C, PDGFB). All qPCR probes were from Thermo Fisher.

### cPLA2 enzymatic activity assay

The cytosolic phospholipase A2 assay kit (Abcam, Cambridge, UK) was used to assess the activity of cPLA2 in treated cells, according to manufacturer’s indications. The samples were concentrated 20 times using Amicon 30 MWCO spin columns (Merck Millipore, Burlington, MS). Samples from three independent experiments were analysed in triplicate. A SpectraMax plate reader (Molecular Devices, San Jose, CA) was used to measure cPLA2 activity.

### Lipidomics

Treated cells were washed twice in cold PBS and then harvested in 100 µl PBS. Medium was collected for ELISA assays and stored at − 80 °C. After centrifugation (1000×*g*, 5 min), the cells were stored at − 80 °C and then submitted to automated homogenization (via ceramic beads) and extraction with BUME method^21^. Internal standards were added to the samples prior to extraction. The lipid extracts were evaporated and reconstituted in chloroform: methanol 2:1 and stored at − 80 °C until analysis. Phospholipids, sphingomyelin, diglycerides, and triglycerides were analyzed by direct infusion mass spectrometry using precursor ion scanning and neutral loss scanning on a QTRAP 5500 mass spectrometer (Sciex, Concord, Ontario Canada) equipped with a TriVersa NanoMate robotic nanoflow ion source (Advion BioSciences, Ithaca, NJ, USA)^22–24^. Sphingolipids were analyzed using ultra performance liquid chromatography coupled to a QTRAP 5500 mass spectrometer as previously described^25^. Data from direct infusion analysis was evaluated using the LipidView software (Sciex, Concord, Ontario, Canada) and lipid levels were calculated by comparing the endogenous signal intensity to the signal from the internal standard signal. Sphingolipids were quantified using external calibration curves.

Five biological replicates were analyzed. Cell count was used as normalizing factor. Briefly, the cell count of a randomly chosen untreated sample was set as reference and its value was set to 1. The cell counts of the remaining 14 samples were compared to the reference, obtaining the respective normalization factors. These factors were used to normalize the lipid measurements. A positive correlation (r = 0.8343, *P* < 0.001) exists between the total lipid amount and the cell count (Supplementary Fig. S1).

### Secretion assays for prostaglandins assessment

Media collected in the lipidomics experiments were assayed for prostaglandins and thromboxanes with competitive ELISA assays. PGE2 was assayed directly (R&D Systems kit) after a 5 min incubation time with the stop solution. PGI2 (prostacyclin) was assessed through its metabolite 6-keto PGF1α and thromboxane A2 (TXA2) through its metabolite thromboxane B2 (TXB2). PGI2 and TXA_2_ kits were from Cayman chemicals, Ann Arbor, MI. Incubation times with the Ellman reagent were 105 min for PGI2 and 120 min for TXB2. Five biological replicates (six for PGE2 assessment after AACOCF3 inhibition) were tested in technical duplicate. The plates were analysed with a SpectraMax plate reader (Molecular Devices).

### Proliferation assays

Mesangial cells were seeded at a concentration of 5000 cells per well in 96 well plates and starved overnight (FBS 0.5%) before performing the BrdU assay (Sigma-Aldrich), following the manufacturer´s method. The luminescence was read after 6 min of incubation with the substrate solution with a SpectraMax plate reader (Molecular Devices). Five independent experiments were conducted, with 8 replicates for each treatment.

### Migration assays

Mesangial cells were seeded in 12 well plates with glass bottom (IBL-Tecan, Männedorf, Switzerland) at a concentration of 100,000 cells per well. The cells were grown until 90% confluence and then starved overnight. Scratches were made with a cell scraper. The cells were washed twice with PBS to remove debris and stimulants were added. Cell migration was analysed with a Cell Discoverer 7 live cell imaging microscope (Zeiss, Oberkochen, Germany). ImageJ 1.53e software was used for the analysis. Five independent experiments were performed, with 3 replicates per each treatment.

### Bio-Plex assays

Cytokines IL-1β and PDGF-BB were quantified with Bio-Plex human cytokine screening assays using the Bio-Plex 200 system (Bio-Rad Laboratories). IL-1β and PDGF-BB were measured in medium after 48 h stimulation with glucose 30 mM, glucose plus mannitol (osmotic control) 30 mM, PDGF-BB 25 ng/ml and IL-1β 1 nM, according to manufacturer’s indications. Stimulation of cells was repeated three times and each medium was then analysed in triplicate.

### Database analysis and validation

The Nephroseq database (https://www.nephroseq.org), which contains patient and animal model gene expression data^26^, was used to validate the regulation of the signaling pathways investigated. The analysed datasets were diabetic nephropathy (human cohorts) and diabetic nephropathy mouse model groups; tissue sources were kidney and glomeruli. Minimum fold change was set at 1.5 and p values considered acceptable when *P* < 0.05. Some genes of interest were included in the validation, although outliers. In these cases, their COPA (Cancer Outlier Profile Analysis) values are plotted (which is analogous to FC, though more speculative in nature)^27^. A recent transcriptomic database (DKD patients versus healthy donor biopsies) was also used in the validation (dataset available on the webpage http://karokidney.org/rna-seq-dn)^28^. All in silico experiments were carried out according to relevant guidelines and regulations. All datasets used were from studies approved by the respective ethics committees, as clearly stated in the referred literature.

### Statistical analyses

Statistical analyses were performed with GraphPad prism Software version 8.3.0 and the R-based tools from LIPID MAPS website, available at https://www.lipidmaps.org/data/stats^29^. IBM SPSS Statistics version 25.0 was used for validation. Sample distribution was tested with Shapiro–Wilk and Kolmogorov–Smirnov tests. Results are presented as mean ± SEM. Differences between 2 groups were assessed with the Mann Whitney test. Differences between 3 or more groups were assessed with the Kruskal–Wallis test and multiple comparison test using the two-stage step-up method of Benjamini, Krieger and Yekuteli (Q = 0.05). Exact p values are given whenever the quantification was possible.

## Results

### High glucose primes IL-1β production; IL-1β and PDGF-BB reciprocally activate their secretion pathways

**Figure 1:**
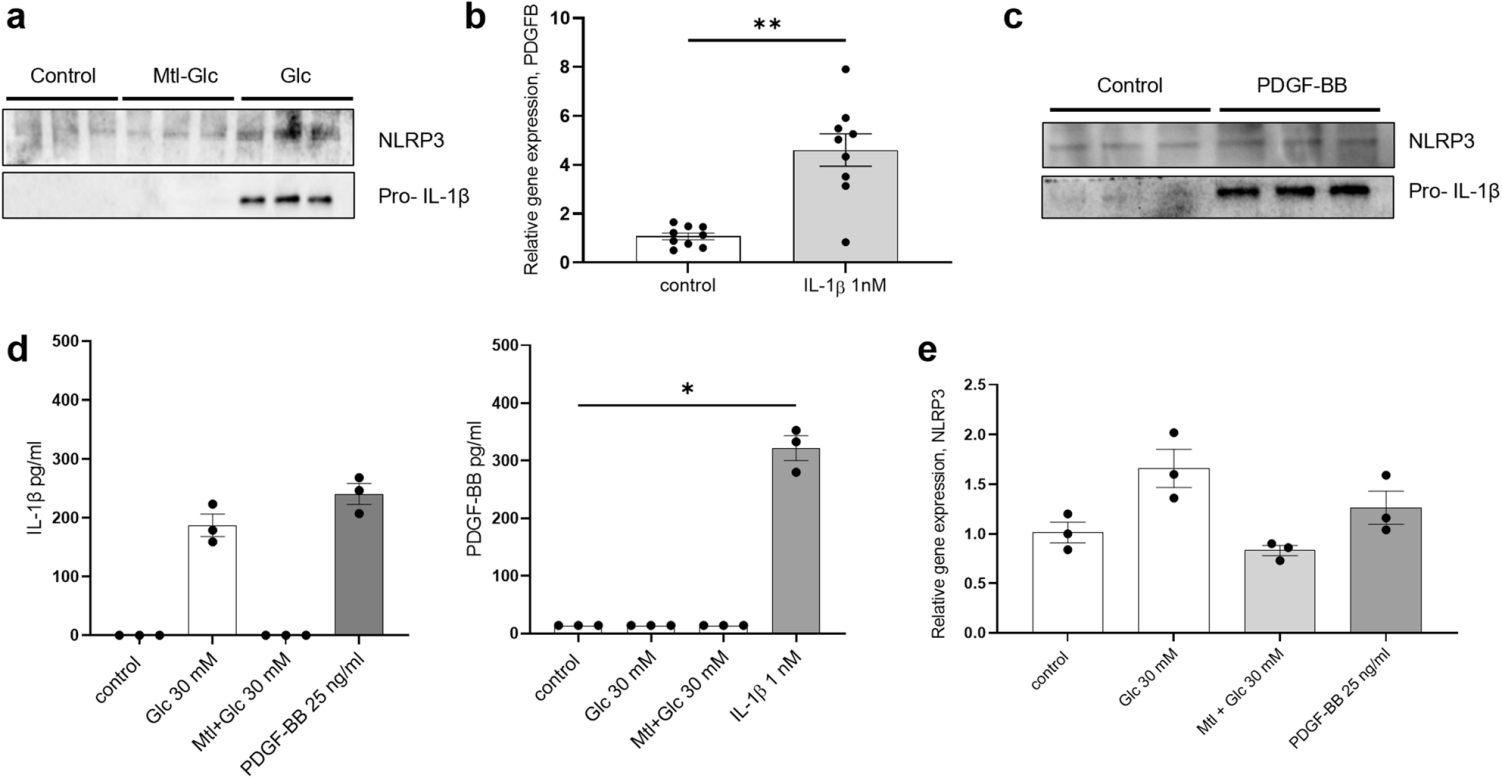
High glucose priming of IL-1β, stimulation of IL-1β on PDGF-BB production, and vice-versa. (**a**) Activation of the inflammasome NLRP3 and subsequent increase of pro-IL-1β protein levels were measured after 24 h stimulation of mesangial cells with Glc 30 mM. Osmotic control was Glc plus mannitol (Mtl), final concentration 30 mM. (**b**) 24 h stimulation of human mesangial cells with IL-1β 1 nM increased the levels of PDGFB mRNA. (**c**) In turn, a 24 h stimulation of mesangial cells with 25 ng/ml of PDGF-BB activated NLRP3 activity and increased pro-IL-1β protein levels. (**d**) Bio-Plex analysis of IL-1β, and PDGF-BB in media after high glucose, osmotic control, IL-1β and PDGF-BB 48 h stimulations. *P < 0.05. Data are reported as mean ± SEM. Samples under the LOD were considered equal to the lowest value in the measurable range (0.25 pg/ml for IL-1β and 13.96 pg/ml for PDGF-BB). (**e**) NLRP3 gene expression levels increased after 24 h stimulation with Glc 30 mM and PDGF-BB 25 ng/ml. Uncropped blots after chemiluminescence development and total protein stain free blots are presented in Supplementary Fig. S4a. Quantification of the bands in the western blot is reported in Supplementary Fig. S5.

To investigate the connection between hyperglycemia and early-stage events in DKD, human mesangial cells were treated for 24 h with high glucose (30 mM) and an osmotic control (normal 17.5 mM glucose plus mannitol, final concentration of 30 mM). Since the formation of the inflammasome NLRP3 and subsequent IL-1β translation has been suggested to be involved in the initiating steps of DKD, their expression was investigated. We found upregulation of NLRP3 (at gene and protein level) as well as of pro-IL-1β protein after high glucose treatment compared to osmotic and untreated controls (Fig. 1a, quantification in Supplementary Fig. S5, NLRP3 gene expression in Fig. 1e).

To further explore the effect of IL-1β, cells were stimulated with IL-1β 1 nM for 24 h. IL-1β stimulation increased PDGFB gene expression compared to control (*P* = 0.001), thus connecting inflammatory stimuli to the production of PDGF-BB, the main proliferative growth factor in mesangial cells (Fig. 1b).

Next, we investigated whether PDGF-BB could activate the inflammasome and IL-1β release, sustaining the pathway. The cells were incubated with PDGF-BB 25 ng/ml for 24 h leading to increased levels of NLRP3 (at gene and protein level) and pro-IL-1β protein (Fig. 1c, quantification in Supplementary Fig. S5, NLRP3 gene expression Fig. 1e).

Bio-Plex analysis were used to quantify IL-1β and PDGF-BB levels in medium. IL-1β secretion was detected after stimulation of mesangial cells with PDGF-BB 25 ng/ml and with glucose 30 mM for 48 h but absent in untreated and osmotic controls. In turn, stimulating mesangial cells with IL-1β 1 nM for 48 h prompted PDGF-BB secretion (*P* = 0.011, Fig. 1d).

These data support the hypothesis of an interplay between IL-1β and PDGF-BB taking place at mesangial level and primed by hyperglycemia.

### IL-1β and PDGF-BB stimulation causes sphingolipids decrease and production of phosphorylated sphingoid bases

**Figure 2:**
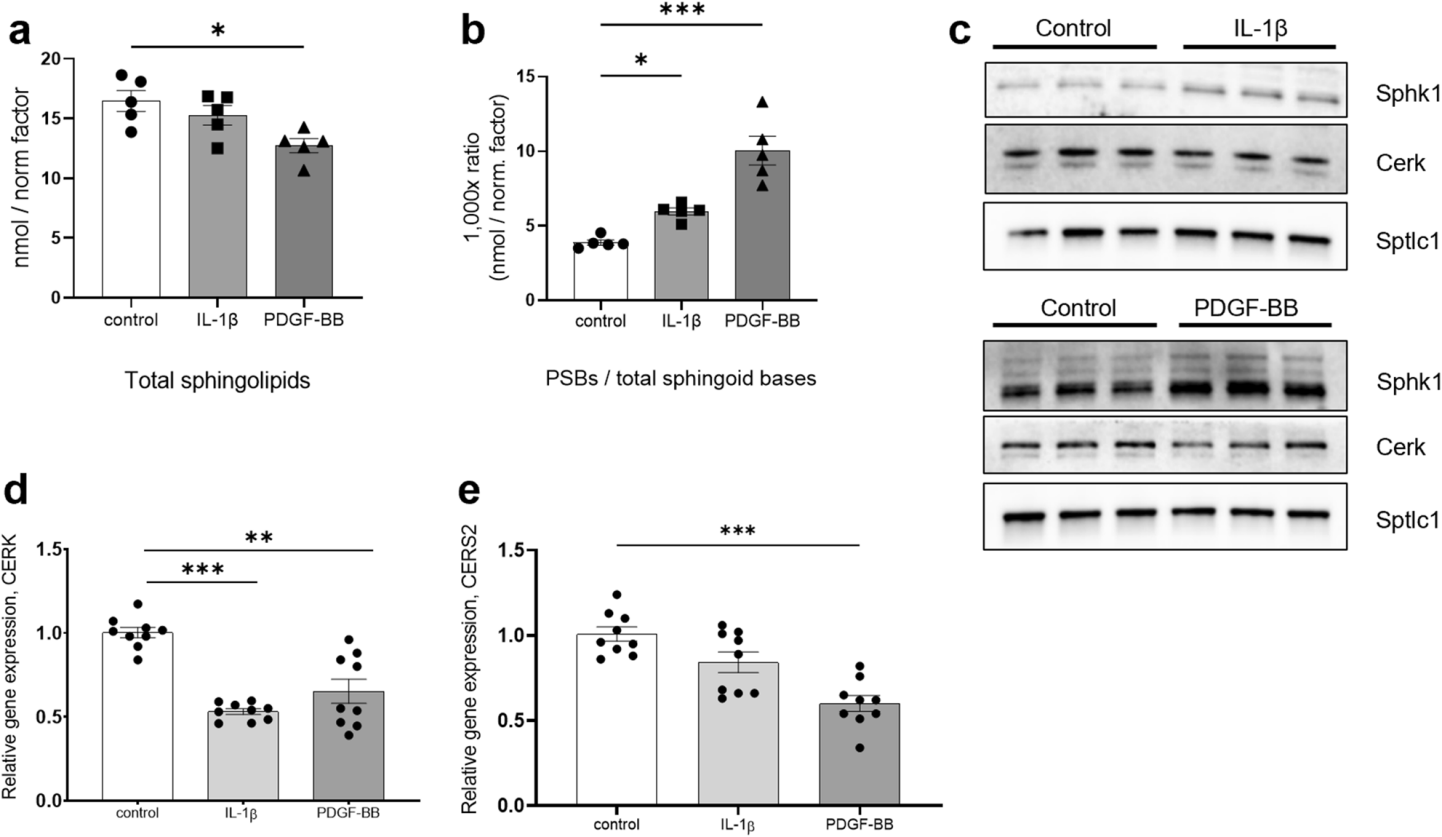
Decreased total ceramides and sphingomyelins and increased phosphorylated sphingoid bases after 24 h stimulation of mesangial cells with IL-1β and PDGF-BB. Lipidomics experiments showed a decrease of the whole sphingolipid cellular pool (Cer, LacCer, dhCer, GluCer, SM, dhSG, SG, S1P, dhS1P, PhytoSG) after the treatments (**a**) and the increase of phosphorylated sphingoid bases (**b**) compared to total sphingoid bases (dhSG, SG, S1P, dhS1P, PhytoSG). Data were normalized against cell count and divided by the relative cell count normalization factor. Sphk1 (phosphorylation of sphingoid bases) protein levels are increased by both stimulations, while Cerk (phosphorylation of ceramides) is downregulated at protein (**c**) and gene level (**d**). SPTLC1 (de novo ceramide synthesis) is not regulated at protein level (**c**) and CERS2 (dhCer synthesis from dhSG) is downregulated at the gene level (**e**). *P < 0.05; **P < 0.01; ***P < 0.001. Data are reported as mean ± SEM. Uncropped blots after chemiluminescence development and total protein stain free blots are presented in Supplementary Fig. S4b. Quantification of the bands in the western blot is reported in Supplementary Fig. S5.

In order to investigate whether lipid signaling might be the missing link between hyperglycemia and mesangial dysfunction in DKD, lipidomic analysis of mesangial cells stimulated with PDGF-BB or IL-1β for 24 h was performed. The results are reported in Supplementary Tables S1–2. The complete lipidomic dataset is reported in Supplementary Tables S3–4. The main finding was a decrease of total amount of sphingolipids in PDGF-BB (*P* = 0.0133) and IL-1β treated cells (ns trend) compared to control (Fig. 2a). Sphingolipid classes such as ceramides (Cer, *P* = 0.0169), glucosylceramides (GluCer, *P* = 0.0039), lactosylceramides (LacCer, *P* = 0.0125) and sphingomyelins (SM, *P* = 0.0140) were all downregulated after PDGF-BB treatment (Supplementary Fig. S2), while no variation was present for dihydroceramides (dhCer, Supplementary Table S1).

Sphingolipids have structural, cell–cell recognition and signaling functions. Among the species that perform the latter function, we observed a variation in the phosphorylated sphingoid bases (PSBs). Briefly, PSBs are obtained through catabolic reactions concluded by the deacylation of unsubstituted ceramides (dhCer and Cer) into sphingosine (SG) or sphinganine (dhSG). Cer, dhCer, SG and dhSG can be phosphorylated respectively by ceramide kinase (Cerk) and sphingosine kinase 1 (Sphk1) to generate signaling sphingolipids. The level of PSBs increased after IL-1β and PDGF-BB treatments (Fig. 2b), when compared to the total sphingoid bases (IL-1β, *P* = 0.0405; PDGF-BB *P* = 0.0004). Sphinganine-1-phosphate (dhS1P) increased after the treatments (PDGF-BB, *P* = 0.0247) and sphingosine-1-phosphate (S1P) remained constant, while non-phosphorylated SG (IL-1β, *P* = 0.0346; PDGF-BB, *P* = 0.0006) and dhSG (IL-1β, *P* = 0.0298; PDGF-BB, *P* = 0.0079) showed a decreasing pattern (Supplementary Fig. S2). Accordingly, Sphk1 protein level increased after treatments (Fig. 2c, quantification in Supplementary Fig. S5), confirming the increased production of PSBs.

Ceramide-1-phosphate (C1P, produced by Cerk) could not be detected with our lipidomic set-up. However, Cerk was decreased at protein and mRNA level (IL-1β, *P* = 0.0004; PDGF-BB, *P* = 0.024) after the treatments (Fig. 2c,d), so C1P is most likely not generated.

To validate the reduction of sphingolipids, the levels of two enzymes were investigated: Serine palmitoyl-transferase 1 (Sptlc1, ubiquitous subunit in the de novo ceramide production complex) and ceramide synthase 2 (Cers2, the most common enzyme responsible for the acylation of dhSG into dhCer in the kidney). If active, Sptlc1 and Cers2 would regenerate the intracellular ceramide pool. Sptlc1 was not regulated at protein level (Fig. 2c). CERS2 gene expression was downregulated by PDGF-BB (*P* < 0.001) and IL-1β (decreasing trend) treatments (Fig. 2e). This confirms that the cellular ceramide pool is not replenished.

Altogether, these results show an intense sphingolipid catabolism leading to generation of PSBs.

### COX-2 activation and prostaglandin secretion

**Figure 3:**
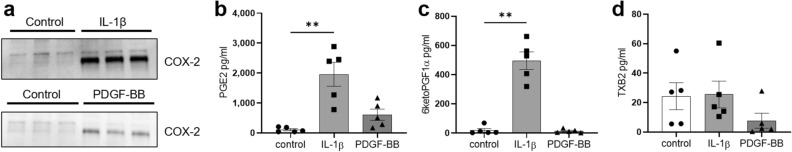
Induction of COX-2 by phosphorylated sphingoid bases and prostaglandins secretion. COX-2 protein levels increased after 24 h stimulation of human mesangial cells with IL-1β or PDGF-BB (**a**). PGE2 is secreted in the medium after both treatments (**b**), while PGI2 (measured through its metabolite 6-keto PGF1α) is secreted after the IL-1β treatment only (**c**). Thromboxane A2 (measured through its metabolite TXB2) secretion is not regulated (**d**). *P < 0.05; **P < 0.01. Data are reported as mean ± SEM. Uncropped blots after chemiluminescence development and total protein stain free blots are presented in Supplementary Fig. S4c. Quantification of the bands in the western blot is reported in Supplementary Fig. S5.

Since PSBs are known to induce COX-2 gene transcription^30^, COX-2 protein expression and downstream secretion of prostaglandins were analysed. COX-2 is the enzyme responsible for transforming arachidonic acid into prostaglandin H2 (PGH2), a precursor molecule for the synthesis of PGs and thromboxanes. COX-2 was upregulated after 24 h stimulation with both IL-1β and (to a lesser extent) PDGF-BB (Fig. 3a).

Prostaglandin PGE2 and PGI2 (assessed by measuring its metabolite 6ketoPGF1α) were both secreted into cell medium after IL-1β treatment (*P* = 0.002, *P* = 0.012, respectively), while PDGF-BB triggered only PGE2 secretion (Fig. 3b,c). None of the treatments induced release of thromboxane A2 (assessed by measuring its metabolite TBX2, Fig. 3d).

Taken together, these data show that stimulation with IL-1β (and to a lesser extent PDGF-BB) activate the secretion of PGs.

### cPLA2s activation provides substrates for COX-2 activity

**Figure 4:**
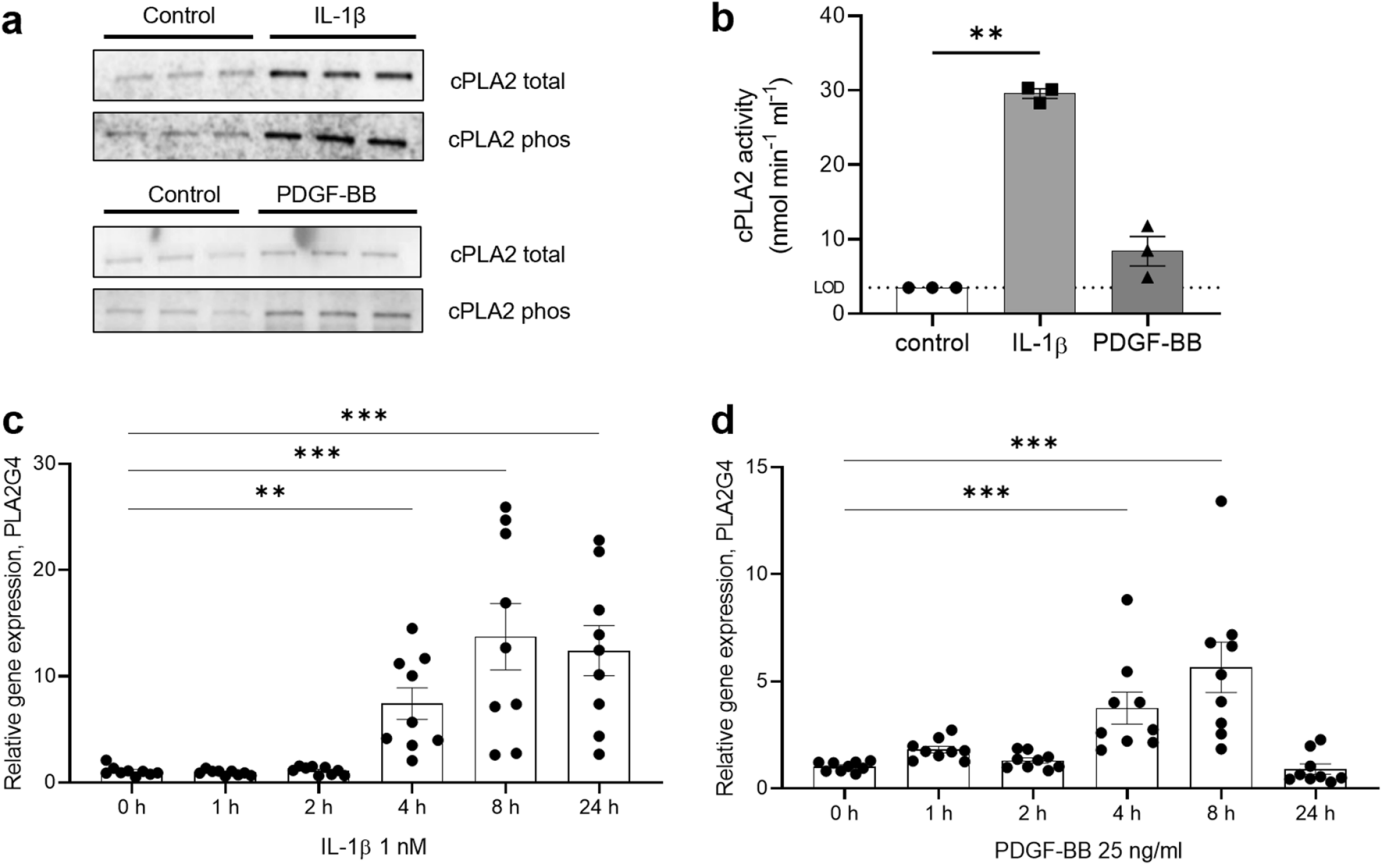
Activation of rate-limiting enzyme cPLA2, upstream of COX-2. Phospholipase cPLA2 is upregulated and phosphorylated in human mesangial cells after 24 h stimulations with IL-1β and PDGF-BB (**a**). cPLA2 activity is accordingly increased (**b**). The activity in the controls was below the LOD of 3.5 nmol/min/ml. In this case, the LOD value was plotted in the graph. PLA2G4A gene expression was already upregulated after 4 h in both treatments (**c**, **d**). *P < 0.05; ***P < 0.001. Data are reported as mean ± SEM. Uncropped blots after chemiluminescence development and total protein stain free blots are presented in Supplementary Fig. S4d. Quantification of the bands in the western blot is reported in Supplementary Fig. S5.

The rate-limiting enzymes of prostaglandin synthesis are cPLA2s, cytosolic phospholipases that produce lysophosphatidylcholine (LPC) and arachidonic acid (substrate for COX-2) from membrane lipids such as phosphatidylcholines (PC). Given the importance of rate-limiting enzymes, the activation of the most abundant cPLA2 (IVA) was analysed in mesangial cells after 24 h treatment with PDGF-BB and IL-1β. Both treatments increased total cPLA2 IVA protein expression and activated the enzyme via phosphorylation (Fig. 4a, quantification in Supplementary Fig. S5). Accordingly, PLA2 activity in both IL-1β (*P* = 0.007) and PDGF-BB treated mesangial cells was increased (Fig. 4b). The activity assay used is not specific to one cPLA2: Arachidonic acid can be released by other mesangial phospholipases, for instance the calcium independent iPLA2 (cPLA2 IVC, PLA2G4C) or the calcium dependent cPLA2 group IVB (PLA2G4B). The expression level of these cPLA2s was found to be upregulated by IL-1β (Supplementary Fig. S6), but at levels not comparable with the increase in the gene expression of cPLA2 IVA (PLA2G4A; *P* < 0.001 after 8 h of both treatments, Fig. 4c,d).

These results show that cPLA2 enzymes are activated in mesangial cells after both IL-1β and PDGF-BB stimulations, with cPLA2 IVA (herein after simply “cPLA2”) being the most upregulated.

### Autotaxin generates lysophosphatidic acid (LPA) from LPC

Although cPLA2 was upregulated and activated, the levels of LPCs did not increase in mesangial cells after 24 h of treatment with IL-1β and PDGF-BB (Supplementary Fig. S3). To elucidate if this could be due to re-acylation of LPCs to form new PCs, the gene expression of the responsible enzymes was investigated. LPCAT1 (LPC acyl transferase) mRNA was not regulated and LPCAT4 mRNA was downregulated (IL-1β, *P* < 0.001, PDGF-BB, *P* = 0.006, Supplementary Fig. S3). Since LPCs are neither accumulated nor recycled by mesangial cells, we hypothesized that they could be either secreted or transformed into LPAs by the enzyme autotaxin (ENPP2). LPCs secretion in cell media was absent (data not shown). Although our lipidomic setup did not allow detection of LPA, a significant upregulation of ENPP2 gene expression levels (IL-1β, *P* = 0.0002; PDGF-BB, *P* = 0.0327) was found in mesangial cells treated for 24 h with IL-1β or PDGF-BB. This is an indirect proof of LPA production from LPC (Supplementary Fig. S3).

These results suggest that LPCs are transformed into LPAs by autotaxin. LPA is known to stimulate proliferation and migration, so it could be considered an effector of PDGF-BB signaling^31–33^.

### Inhibition of cPLA2 with AACOCF3 reduces cell proliferation, migration and prostaglandin secretion

The expansion of the mesangium is one of the key findings in DKD and PDGF-BB is the main proliferative signaling cytokine for mesangial cells. Keeping this in mind, we went on to explore if cPLA2 could be involved in the regulation of mesangial proliferation and migration through cPLA2 activation, secretion of LPA and, possibly, PGs.

As shown before, PDGF-BB stimulates cPLA2 activity and PGs secretion directly and indirectly through IL-1β production. Glomerular hemodynamic alterations are early-stage findings in DKD and can be ascribed to PGE2 (and PGI2) secretion. Hence, we speculated whether cPLA2 inhibition could be sufficient to reduce PDGF-BB induced proliferation and migration, and even the PGs production induced by PDGF-BB and IL-1β.

**Figure 5:**
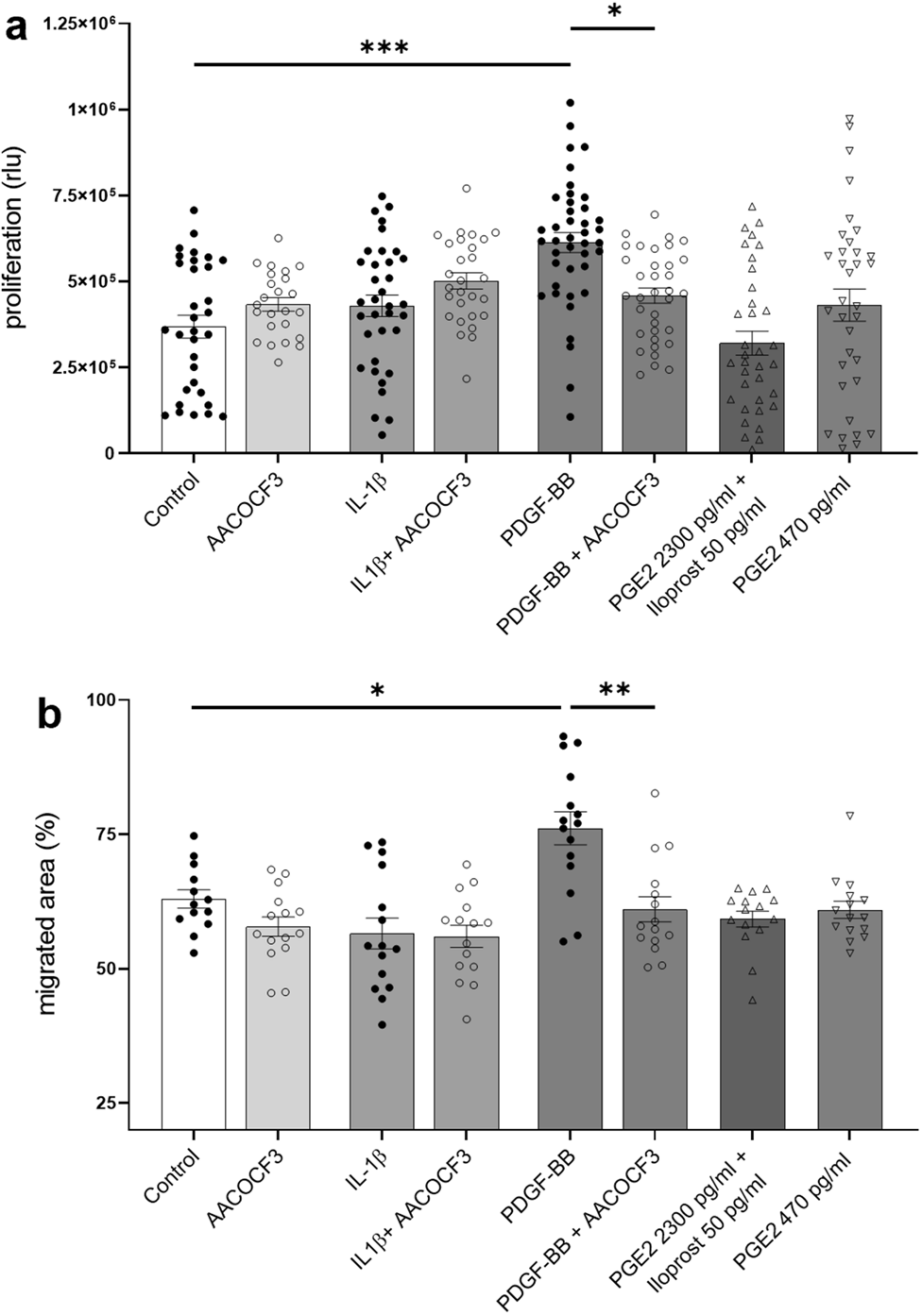
Inhibition of cPLA2 with AACOCF3 1 μM reduces proliferation, secretion, and prostaglandin secretion. AACOCF3 reduced PDGF-BB driven cell proliferation (**a**) and migration (**b**). No effect was observed when PGE2 and iloprost (PGI2) were administered together (mimicking their secretion levels obtained after 24 h IL-1β stimulation of mesangial cells) and at low PGE2 concentrations (mimicking PGE2 secretion levels after 24 h PDGF-BB stimulation) (**b**). Prostaglandin administration had no migratory effect on mesangial cells (**b**). *P < 0.05; **P < 0.01; ***P < 0.001, *rlu* relative light units. Data are reported as mean ± SEM.

Proliferation and migration assays were performed by treating mesangial cells for 24 h with PDGF-BB and IL-1β with or without pre-treatment with cPLA2 inhibitor AACOCF3 for 1 h. Mesangial cells stimulated with PDGF-BB proliferated (*P* < 0.001) and migrated (*P* = 0.034) as expected. IL-1β did not affect neither proliferation nor migration, when compared to untreated cells. Blocking cPLA2 using AACOCF3 significantly reduced both the proliferative and migratory effects of PDGF-BB (*P* = 0.02 and *P* = 0.01 respectively) (Fig. 5a,b).

Neither PGE2 administered alone (470 pg/ml, representing the secretion of PGE2 after PDGF-BB stimulation) nor in combination with iloprost (2300 pg/ml of PGE2 and 50 pg/ml of iloprost, representing PGs secretion after IL-1β stimulation) affected mesangial proliferation and migration (Fig. 5a,b). The concentrations used were based on average secretions calculated from earlier experiments (data not shown). This excludes a mesangio-proliferative activity of prostaglandins.

**Figure 6:**
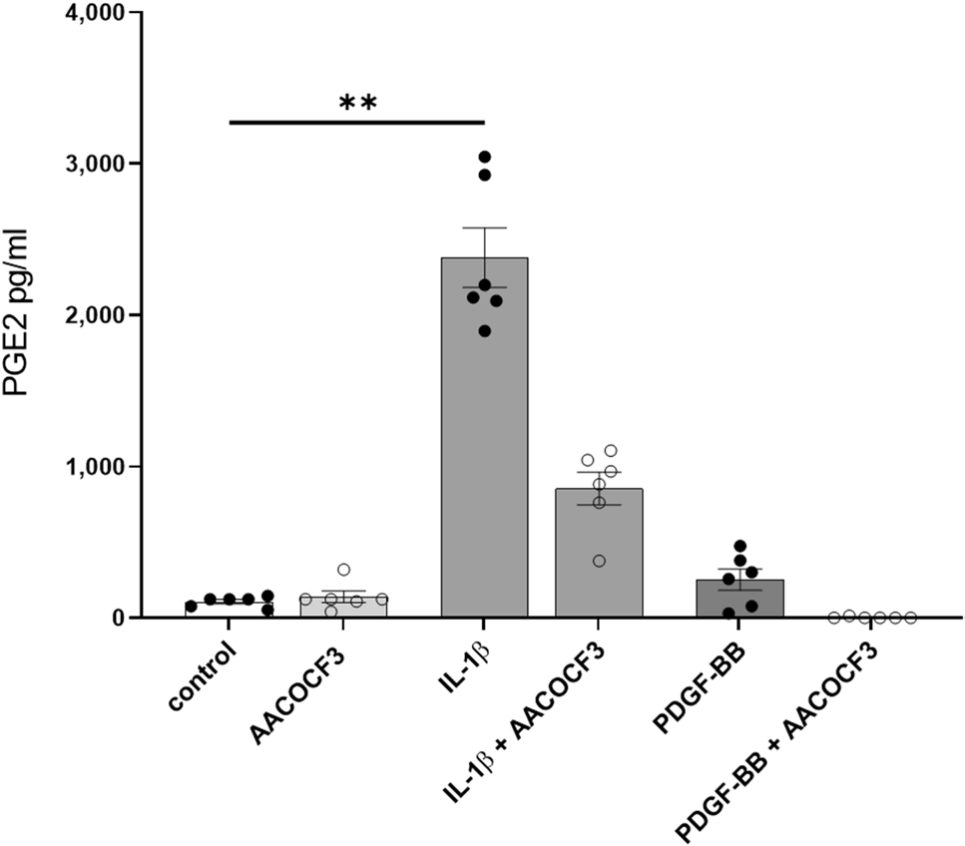
Inhibition of cPLA2 with AACOCF3 1 μM reduces IL-1β, PDGF-BB induced PGE2 secretion. **P < 0.01. Data are reported as mean ± SEM.

Moreover, AACOCF3 was effective in reducing the production of PGE2 in pre-treated mesangial cells by reducing the availability of the COX-2 substrate arachidonic acid (*P* = 0.007, Fig. 6). This once again underlined the importance of cPLA2 in the pathway.

Altogether, the data collected indicate that inhibition of cPLA2 might be able to reduce early events in the development of DKD, namely mesangial expansion and the alterations in glomerular hemodynamics by prostaglandins.

### Data validation in human and murine cohorts

The transcription profiles of cPLA2, ceramide metabolism enzymes, COX-2, Sphk1 and PG and LPA receptors were investigated using the Nephroseq database and in particular glomerular data from DKD and diabetic animal models and DKD patients^34–36^. Additional validation was obtained using a recently published DKD patient glomerular mRNA deep sequencing data^28^.

**Figure 7:**
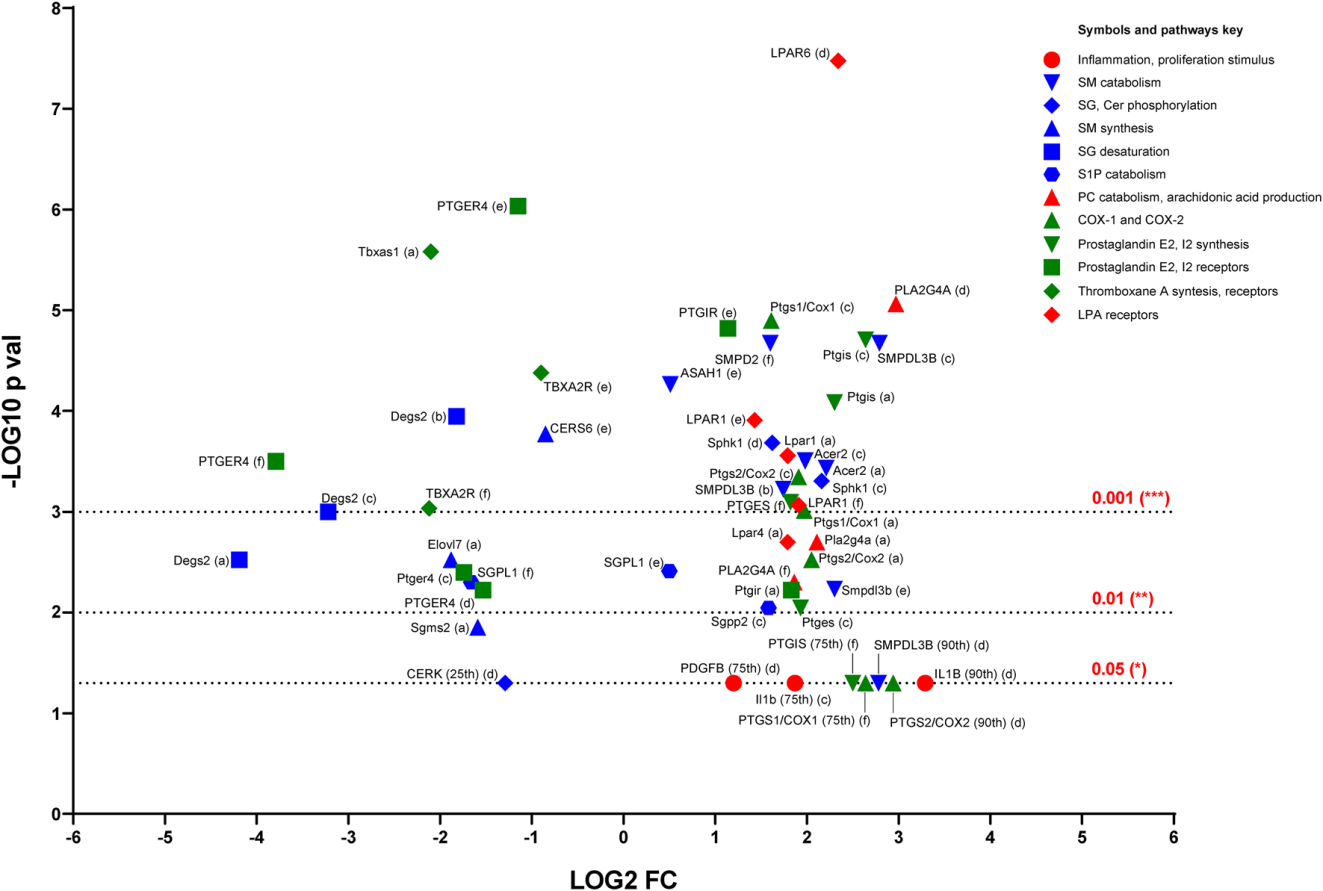
Volcano plot of the in silico data validation performed using glomerular gene expression data from Nephroseq and glomerular mRNA sequencing data from Levin et al.^28^. Human gene names are reported in uppercase, mouse genes names in lowercase. (**a**) Hodgin et al.^34^ db/db C57BLKS mouse model—DKD vs non-DKD. (**b**) Hodgin et al., eNOS-deficient C57BLKS db/db mouse model—DKD vs non-DKD. (**c**) Hodgin et al., DBA/2 mouse model—DKD vs non-DKD. (**d**) Ju et al.^35^ DKD human renal biopsies vs healthy individuals. (**e**) Levin et al.^28^ DKD human renal biopsies vs healthy individuals. (**f**) Woroniecka et al.^36^ DKD human renal biopsies vs healthy individuals. For the outliers, the relative percentiles were reported after the gene names and. COPA values were plotted instead of log2 fold changes.

The analysis confirmed the experimental data. We found upregulated gene expression of SPHK1, cPLA2, COX-2, PG synthases, PG receptors, LPA receptors, and enzymes responsible for sphingolipid catabolism (ceraminidases ACER2 and ASAH1, sphingomyelin phosphodiesterases SMPD2 and SMPDL3B). In addition, sphingolipid synthesis (Sphingomyelin synthase SGMS, ceramide synthase CERS, elongase VL) was decreased, while PSBs catabolism (lyase SGPL1, phosphatase SGPP2) showed an inconclusive trend (Fig. 7, Supplementary Table S5).

## Discussion

Inflammation and expansion of the mesangium are key events in DKD and lead to proteinuria, renal dysfunction and in the longer perspective loss of function. We have found that hyperglycemia, via a synergistic interplay of inflammatory (IL-1β) and proliferative stimuli (PDGF-BB), alters lipid metabolism and stimulates mesangial proliferation and release of PGs and LPAs via the cPLA2-COX2 pathway.

cPLA2 group IVA was found to be a key enzyme in these events. The involvement of other cPLA2 enzymes cannot be excluded, as both the activity assay used and the inhibitor are not targeting a single enzyme but rather the entire cPLA2 enzymatic pool. Still, the contribution is probably limited, given the difference in upregulation that we have shown.

**Figure 8:**
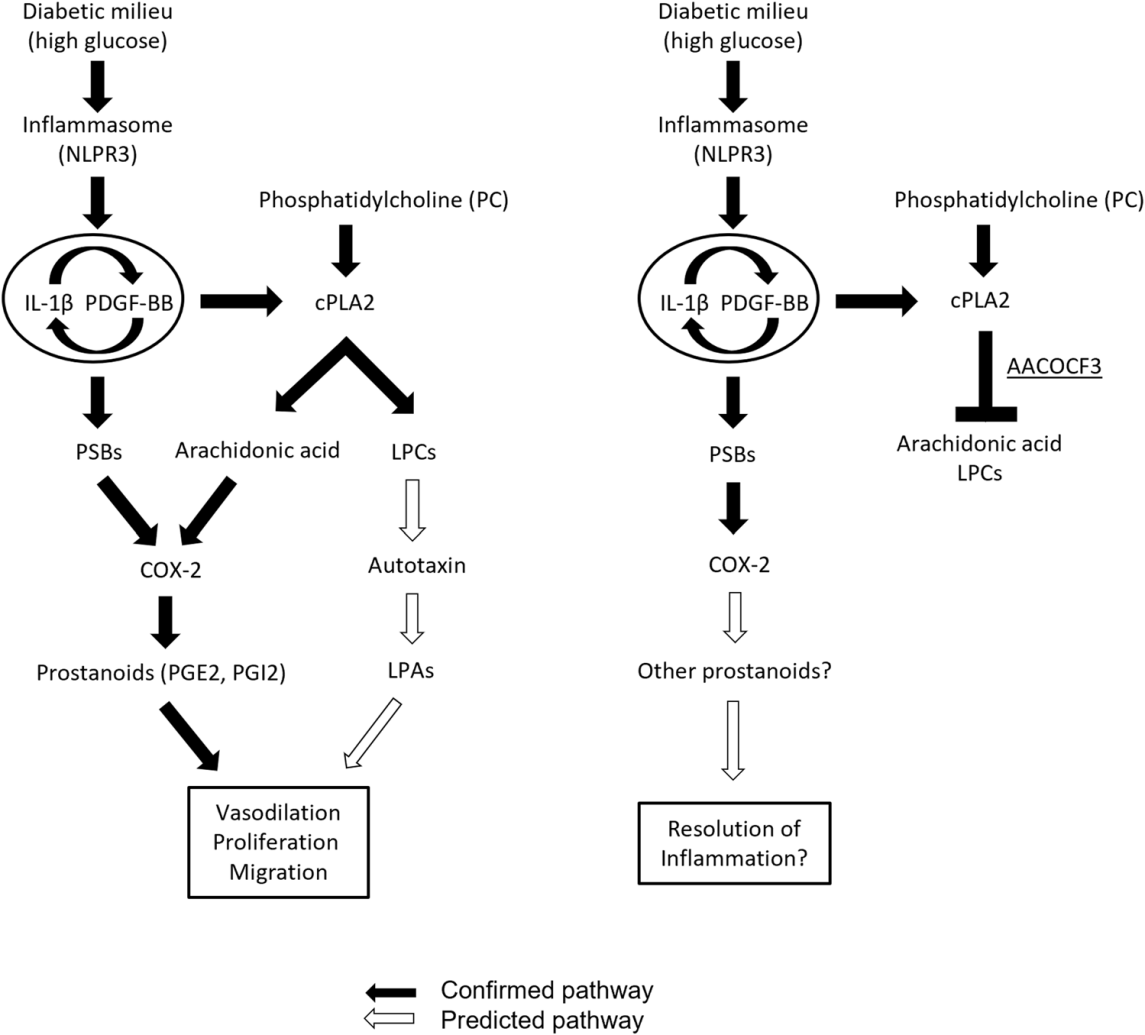
Signaling pathway overview. Hyperglycemia stimulates mesangial production of IL-1β which activates PDGF-BB secretion. In turn, PDGF-BB promotes IL-1β secretion, sustaining and boosting the pathway activation. IL-1β and PDGF-BB stimulation give rise to the production of phosphorylated sphingoid bases, activating COX-2 transcription. At the same time, IL-1β and PDGF-BB stimulate cPLA2 activation. Arachidonic acid (AA) released by cPLA2 is converted into prostanoids by COX-2 and downstream enzymes. The other product of cPLA2, lysophosphatidylcholine (LPC), is converted into lysophosphatidic acid (LPA) by autotaxin. Thus, hyperglycemia triggered the activation of an interplay between IL-1β and PDGF-BB which stimulates the secretion of lipid hormones (prostanoids PGE2, PGI2, but also lysophosphatidic acid) with hemodynamic, proliferative, and migratory effects at glomerular level. Inhibition of the cPLA2 reaction with AACOCF3 blocks the supply of AA for the COX-2 reaction, thus resolving the inflammatory stimulus. In addition, LPC is not produced, and this blocks the supply for the autotaxin reaction and LPA mediated proliferative response.

Reduced prostaglandin secretion as well as reduced PDGF-BB driven proliferation and migration were obtained when blocking cPLA2. Though limited by the absence of an in vivo model to support our cell-based investigation, our findings were confirmed by in silico validation, obtained by using publicly available data from human DKD cohorts as well as animal models. The suggested signaling pathway is schematized in Fig. 8.

To our knowledge, a functional synergy between IL-1β and PDGF-BB has so far not been investigated at mesangial level. In vitro studies of mesangial cells have shown that IL-1β can induce a delay in PDGF-BB and PDGF-AB mediated growth response^39^. Conversely, in our study we found that PDGF-BB expression is upregulated by IL-1β stimulation and secreted into cell medium. In addition, we found that PDGF-BB activates the inflammasome driven expression and secretion of IL-1β and that both IL-1β and PDGF-BB are able to upregulate cPLA2 and COX-2 signaling, driving prostaglandin production.

Lipidomic analysis of IL-1β and PDGF-BB treated mesangial cells revealed reduction of sphingolipids and production of PSBs. Increased levels of S1P have been reported in plasma from type 1 diabetes patients compared to healthy individuals^40^, and the levels of circulating dhS1P were found to be associated with increasing risk for type 2 diabetes^41^. In other words, an increase in production of PSBs could precede DKD onset.

Earlier studies by other groups have shown that high glucose activates inflammasome NLRP3 triggering IL-1β expression and activation in vivo in diabetic mouse and rat models and in cultured mesangial cells^10,11,37^. Furthermore, inflammasome activation has been linked to increased prostaglandin production^38^, therefore being of interest in the priming of DKD.

PSBs are short-lived metabolites, not accumulated but instead rapidly cleared by degradative enzymes^42,43^. This is probably the reason why we could not detect an increase of S1P. Nevertheless, Sphk1 upregulation supports the probability of S1P overproduction.

The PSBs generated in the stimulated mesangial cells caused COX-2 induction, in turn triggering prostaglandin release. It has been reported that COX-2 gene levels are upregulated in rat mesangial cells treated with PDGF-BB without any demonstrated prostaglandin connection^44^. In our study, both IL-1β and PDGF-BB stimulation gave rise to the production of PGE2, in agreement with what has been discovered earlier in rat mesangial cells^45^. The release of prostaglandins might cause afferent arteriolar vasodilation, leading to hyperfiltration, and to an irreversible dysfunction of the nephron^2^. These hemodynamic variations are known symptoms of early stage DKD and the modulation of vasodilatory events has indeed been suggested as a target for DKD treatment^46^.

PGE2 is generally considered the most important vasodilator PG in the kidney, although its role in DKD is not fully understood^47,48^. Interestingly, blockade of the main PGE2 glomerular receptor (EP1) has been found beneficial against mesangial expansion in vitro in mouse cells and in vivo in mouse and rat models^49,50^. The production of PGI2 after IL-1β stimulus should increase PGE2 driven vasodilation through IP receptor signaling^51^. In agreement, secretion of the vasoconstrictor thromboxane A2 was not detected after stimulation with IL-1β and PDGF-BB^52^.

Stimulation of mesangial cells with PDGF-BB induced proliferation, while pretreatment of the cells with the cPLA2 inhibitor AACOCF3 blocked PDGF-BB proliferative effect on the cells. Furthermore, PDGF-BB stimulus increased migration and this effect was blocked by AACOCF3 as well. Mesangial cells migration and proliferation are key findings in many glomerular diseases including DKD. The fact that blocking cPLA2 interferes with both PDGF-BB migratory and proliferative response indicates that cPLA2 has a major role in controlling downstream PDGF signaling in mesangial cells. Moreover, inhibition of cPLA2 blocked PGE2 secretion and might therefore prevent vasodilation as well, though this can only be confirmed with an in vivo model.

We suggest that the link between cPLA2 and mesangial cells proliferation could be LPA, generated from LPC by autotaxin. Mesangial cells are known to secrete LPA and express LPA receptors. Urinary LPA levels are increased in DKD patients and in DKD murine models^53–56^. Although it was not possible to measure LPA in our lipidomics setup, autotaxin (ENPP2) gene expression levels were increased after IL-1β and PDGF-BB stimuli, thus the enzyme that produces LPA is upregulated. LPC remodeling enzymes LPCAT1 and 4 were downregulated, so clearance of LPC could not be achieved by regenerating PCs. Taken together, this points towards secretion of LPA which could amplify or convey PDGF-BB signaling promoting, proliferation^31–33^.

While the activation of cPLA2 was stronger in the IL-1β treated cells, the decrease of sphingolipids was more pronounced in the PDGF-BB treated cells. We have found that IL-1β triggers an initial activation of cPLA2, COX-2 and prostaglandin synthases. Since IL-1β stimulates PDGFB gene expression as well as PDGF-BB secretion, the pathway is further amplified and sustained, leading to changes in lipid metabolism. Moreover, PDGF-BB keeps the cascade ongoing by stimulating mesangial cells to produce and secrete more IL-1β. This interplay will most probably lead to pathological crosstalk between the glomerular cells further driving the progression of DKD.

As of today, there is no curative treatment for DKD, and treatment strategies aim mainly at handling the diabetic preexistent condition to delay DKD progression. Targeting IL-1β signaling in diabetes has been suggested and treatments targeting PDGF signaling has also been proposed for pathologies involving mesangial proliferation, for instance IgA Nephropathy^7,9,57–59^. The results from our study show that inhibition of cPLA2 reduces the effects of both inflammatory and proliferative stimuli by simultaneously blocking the secretion of PGs and LPA. Thus, modulation of cPLA2 could be considered as a potentially more specific target for early treatment and prevention of DKD and other mesangioproliferative diseases.

## Supplementary Information

Supplementary Information.

## Data Availability

All data generated or analysed during this study are included in the manuscript article (and Supplementary Tables S1–4). The data that support the findings of this study are freely available at http://karokidney.org/rna-seq-dn^28^ and at https://www.nephroseq.org^26^.
