# Supplementary material for: Modified lipid metabolism and cytosolic phospholipase A2 activation in mesangial cells under pro-inflammatory conditions
Source: Sci Rep. 2022 May 5;12:7322. doi: 10.1038/s41598-022-10907-4 (PMC9072365; doi:10.1038/s41598-022-10907-4)
Supplement: Supplementary file 1 — Supplementary Information. [file 41598_2022_10907_MOESM1_ESM.pdf]

Supplementary table of contents

Modified lipid metabolism and cytosolic phospholipase A2 activation in mesangial cells under pro-inflammatory conditions

Roberto Boi, Kerstin Ebefors, Marcus Henricsson, Jan Borén, and Jenny Nyström.

Supplementary Figures Legends

Supplementary Figure S1.

Lipidomics data normalization and correlation between cell count and total lipids amount. Spearman nonparametric correlation R and a linear regression between the three data cohorts is plotted. *** $P<0.001$. Total lipid levels, cell count and normalization factors per each sample are reported in the table. Lipidomics data were divided by the relative cell count normalization factor.

Supplementary Figure S2.

Treatment with IL-1β and PDGF-BB influences sphingolipids metabolism. Ceramides (a), glucosylceramides (B), lactosylceramides (C) and sphingomyelins (D) were reduced after PDGF-BB treatment. Phosphosphingosine S1P was not regulated (E), dhS1P increases (F) after the treatments. Sphingosine (G) and sphinganine (H) decreased in both treatments.

* $P<0.05$; ** $P<0.01$; *** $P<0.001$. Data were normalized against cell count and divided by the cell count relative normalization factor and are reported as mean ± SEM.

Supplementary Figure S3.

Lysophosphatidylcholines are transformed in lysophosphatidic acid by autotaxin. Lysophosphatidylcholine class was not upregulated after 24h IL-1β and PDGF-BB treatments. Data were normalized against cell count and divided by the relative normalization factor (A). Land´s remodeling cycle enzymes are either not (LPCAT1, panel B) or downregulated (LPCAT4, panel C) at gene level. ENPP2 (autotaxin) gene expression is upregulated after IL-1β and PDGF-BB treatments (D). * $P<0.05$; *** $P<0.001$. Data are reported as mean ± SEM.

Supplementary Figure S4.

a) Full blots and relative total protein blots used for normalizations, as in Figure 1a and 1c. First lane was always loaded with All Blue Precision Plus stained ladder; the second lane with unstained MW ladder (identical MW, Bio-Rad). Red boxes correspond to cropped blots reported in the relative panels. The same total protein blot was used for both NRLP3 and pro-IL-1β in Figure 1c. The total protein blot was divided and incubated separately with the respective antibodies.

b) Full blots and relative total protein blots used for normalizations, as in Figure 2c. First lane was always loaded with All Blue Precision Plus stained ladder; the second lane with unstained MW ladder (identical MW, Bio-Rad). Red boxes correspond to cropped blots reported in the relative panels.

c) Full blots and relative total protein blots used for normalizations, as in Figure 3a. First lane was always loaded with All Blue Precision Plus stained ladder; the second lane with unstained MW ladder (identical MW, Bio-Rad). Red boxes correspond to cropped blots reported in the relative panels.

d) Full blots and relative total protein blots used for normalizations, as in Figure 4a. First lane was always loaded with All Blue Precision Plus stained ladder; the second lane with unstained MW ladder (identical MW, Bio-Rad). Red boxes correspond to cropped blots reported in the relative panels. Some of the total protein blots presented here are the same used for quantifications in Supplementary Figure S4b. The total protein blot was divided and incubated separately with the respective antibodies.

Supplementary Figure S5.

Protein band quantification for each of the blots in Figures 1-4. Total protein lane content was normalized and quantification obtained using the stain free total protein method.

Supplementary Figure S6.

Gene expression analysis of two other PLA2 present in human mesangial cells, PLA2G4B (cPLA2 group IVB) and PLA2G4C (cPLA2 group IVC or iPLA2).

Supplementary Tables

Supplementary Table S1.

Lipidomic analysis. Results for lipid classes analysis.

Supplementary Table S2.

Lipidomic analysis. Results for each lipid species measured.

Supplementary Table S3.

Lipidomic data. Results for each lipid class measured, normalized nmol amounts.

Supplementary Table S4.

Lipidomic data. Results for each lipid species measured, normalized nmol amounts.

Supplementary Table S5.

Data validation performed in silico using glomerular gene expression data from Nephroseq and glomerular mRNA sequencing data from Levin et al.

Supplementary Figure S1.

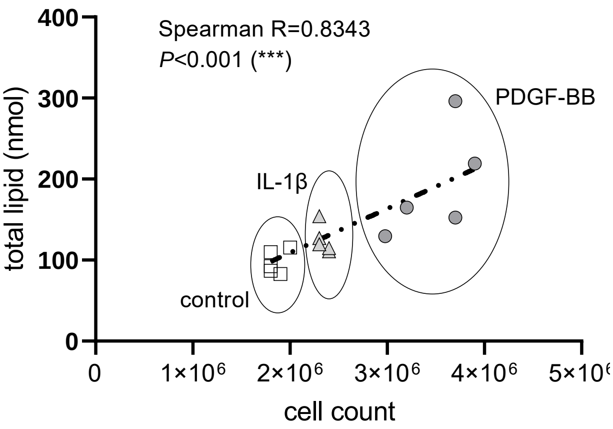

Sample	Total lipids (nmol per sample)	Cell count	Normalization factor (cell count)
control 1	82.83	1.90E+06	1.00
control 2	92.54	1.80E+06	0.95
control 3	86.78	1.80E+06	0.95
control 4	109.75	1.80E+06	0.95
control 5	115.38	2.00E+06	1.05
IL-1β 1	154.28	2.30E+06	1.21
IL-1β 2	127.15	2.30E+06	1.21
IL-1β 3	110.77	2.40E+06	1.26
IL-1β 4	114.88	2.40E+06	1.26
IL-1β 5	119.45	2.30E+06	1.21
PDGF-BB 1	152.56	3.70E+06	1.95
PDGF-BB 2	129.49	2.98E+06	1.57
PDGF-BB 3	219.19	3.90E+06	2.05
PDGF-BB 4	164.78	3.20E+06	1.68
PDGF-BB 5	296.06	3.70E+06	1.95

Supplementary Figure S1. Lipidomics data normalization and correlation between cell count and total lipids amount. Spearman nonparametric correlation R and a linear regression between the three data cohorts is plotted. *** $P<0.001$. Total lipid levels, cell count and normalization factors per each sample are reported in the table. Lipidomics data were divided by the relative cell count normalization factor.

Supplementary Figure S2.

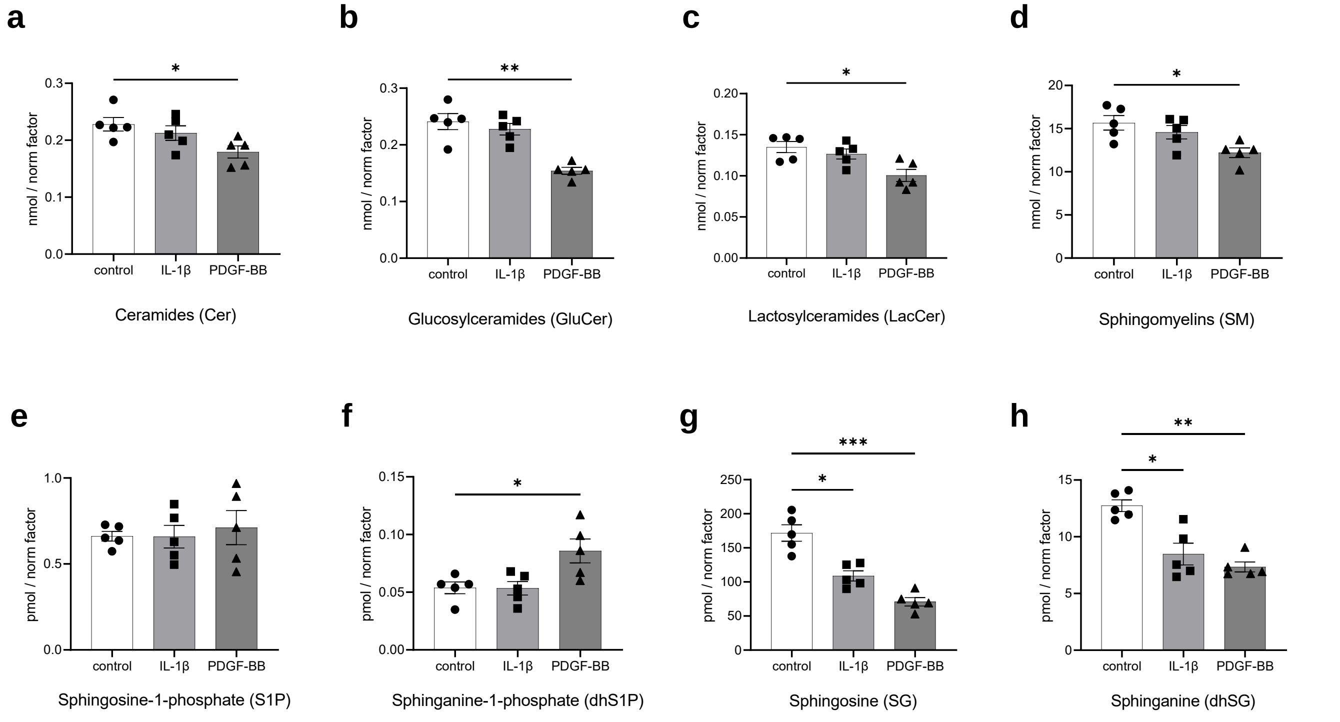

Supplementary Figure S2. Treatment with IL-1β and PDGF-BB influences sphingolipids metabolism. Ceramides (a), glucosylceramides (B), lactosylceramides (C) and sphingomyelins (D) were reduced after PDGF-BB treatment. Phosphosphingosine S1P was not regulated (E), dhS1P increases (F) after the treatments. Sphingosine (G) and sphinganine (H) decreased in both treatments. * $P<0.05$; ** $P<0.01$; *** $P<0.001$. Data were normalized against cell count and divided by the cell count relative normalization factor and are reported as mean ± SEM.

Supplementary Figure S3.

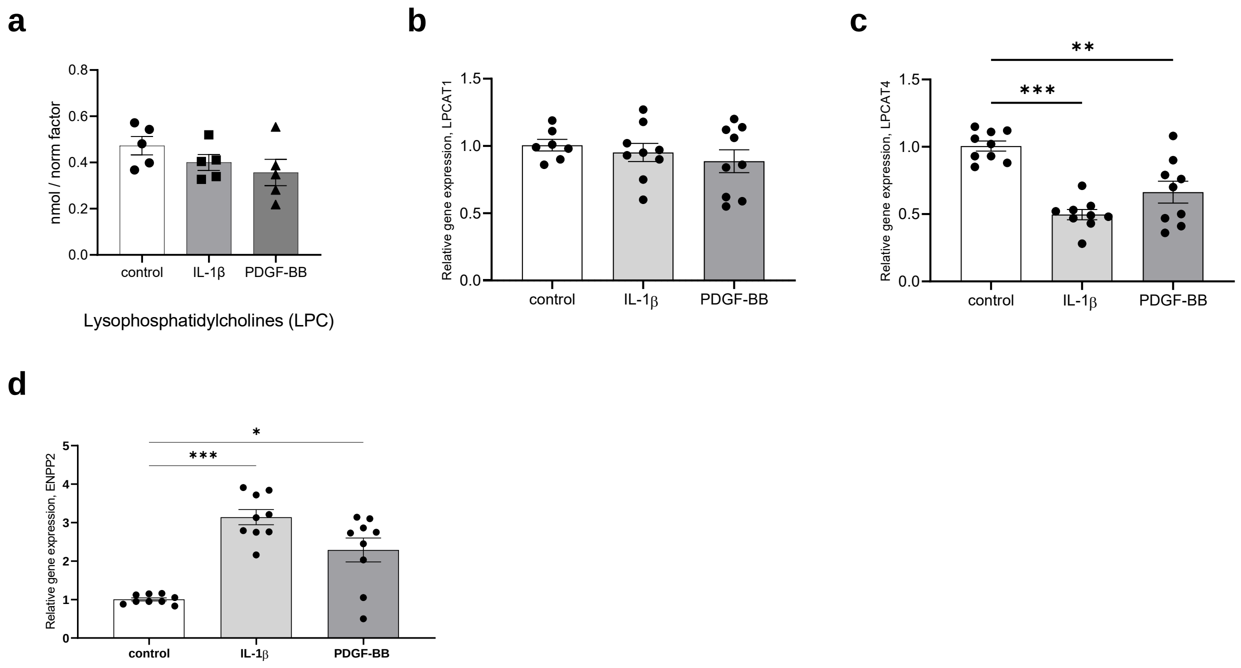

Supplementary Figure S3. Lysophosphatidylcholines are transformed in lysophosphatidic acid by autotaxin. Lysophosphatidylcholine class was not upregulated after 24h IL-1β and PDGF-BB treatments. Data were normalized against cell count and divided by the relative normalization factor (A). Land´s remodeling cycle enzymes are either not (LPCAT1, panel B) or downregulated (LPCAT4, panel C) at gene level. ENPP2 (autotaxin) gene expression is upregulated after IL-1β and PDGF-BB treatments (D). * P<0.05; *** P<0.001. Data are reported as mean ± SEM.

Supplementary Figure S4a.

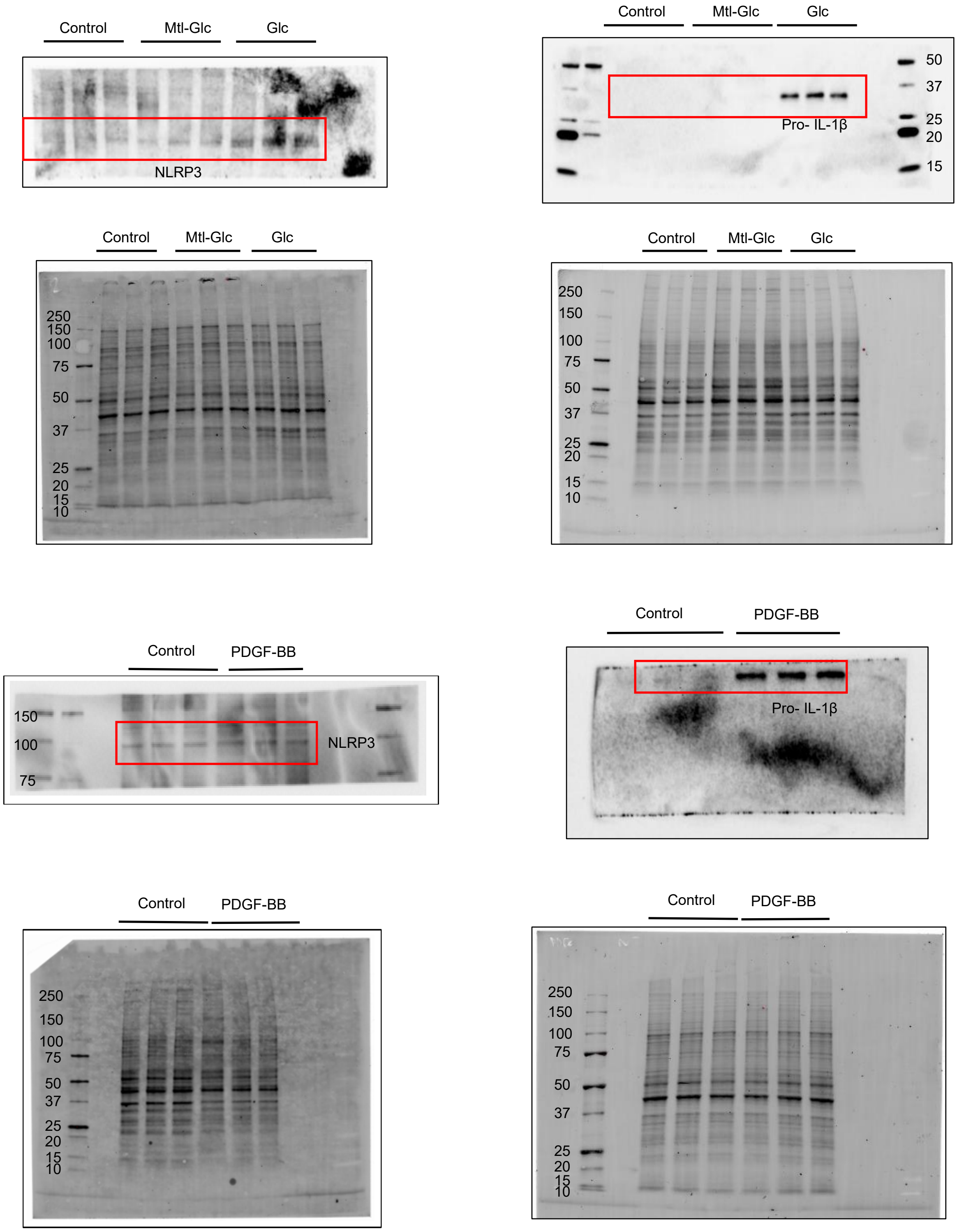

Supplementary Figure S4a. Full blots and relative total protein blots used for normalizations, as in Figure 1a and 1c. First lane was always loaded with All Blue Precision Plus stained ladder; the second lane with unstained MW ladder (identical MW, Bio-Rad). Red boxes correspond to cropped blots reported in the relative panels. The same total protein blot was used for both NRLP3 and pro-IL-1β in Figure 1c. The total protein blot was divided and incubated separately with the respective antibodies.

Supplementary Figure S4b.

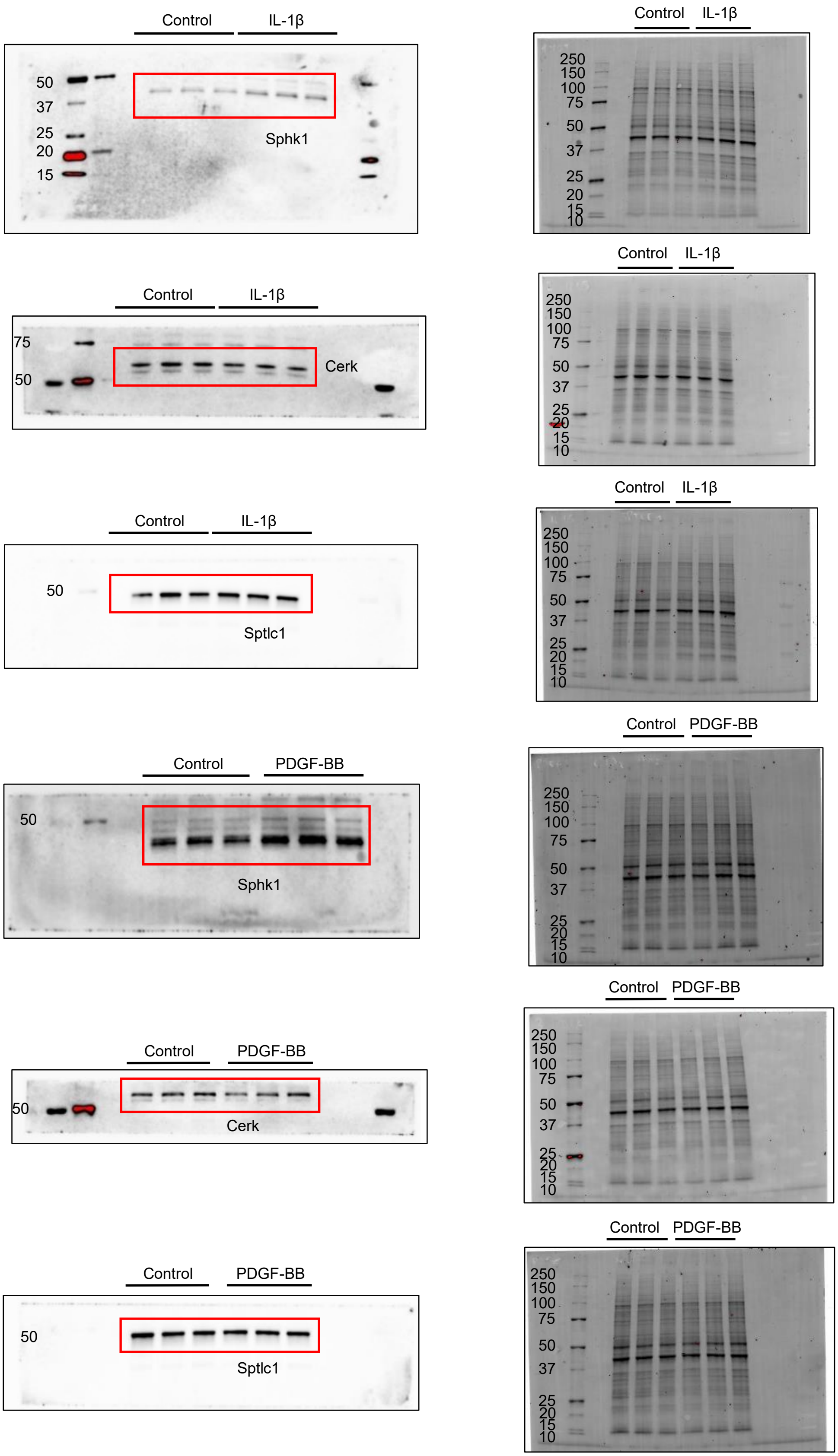

Supplementary Figure S4b. Full blots and relative total protein blots used for normalizations, as in Figure 2c. First lane was always loaded with All Blue Precision Plus stained ladder; the second lane with unstained MW ladder (identical MW, Bio-Rad). Red boxes correspond to cropped blots reported in the relative panels.

Supplementary Figure S4c.

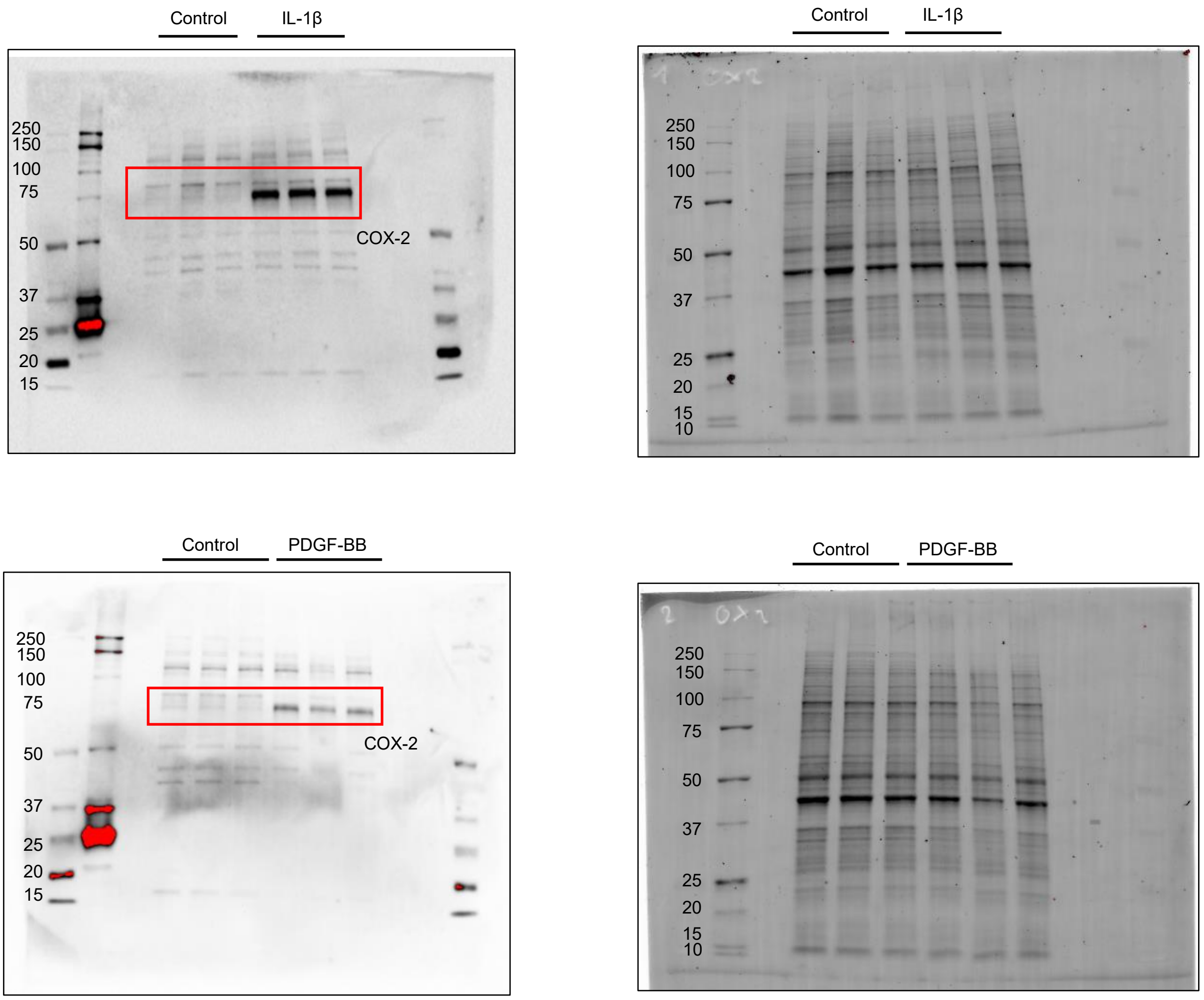

Supplementary Figure S4c. Full blots and relative total protein blots used for normalizations, as in Figure 3a. First lane was always loaded with All Blue Precision Plus stained ladder; the second lane with unstained MW ladder (identical MW, Bio-Rad). Red boxes correspond to cropped blots reported in the relative panels.

Supplementary Figure S4d.

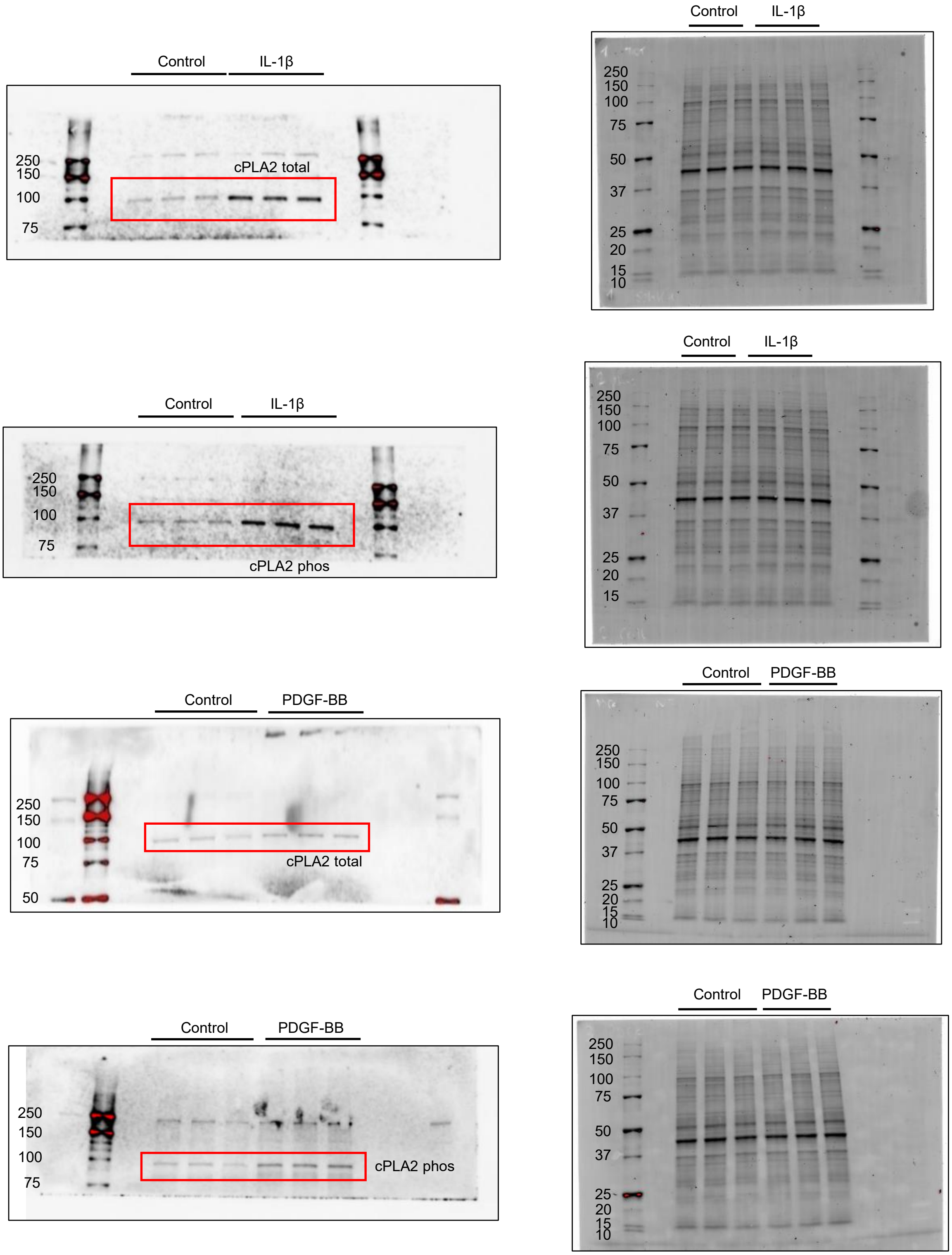

Supplementary Figure S4d. Full blots and relative total protein blots used for normalizations, as in Figure 4a. First lane was always loaded with All Blue Precision Plus stained ladder; the second lane with unstained MW ladder (identical MW, Bio-Rad). Red boxes correspond to cropped blots reported in the relative panels. Some of the total protein blots presented here are the same used for quantifications in Supplementary Figure S4b. The total protein blot was divided and incubated separately with the respective antibodies.

Supplementary Figure S5.

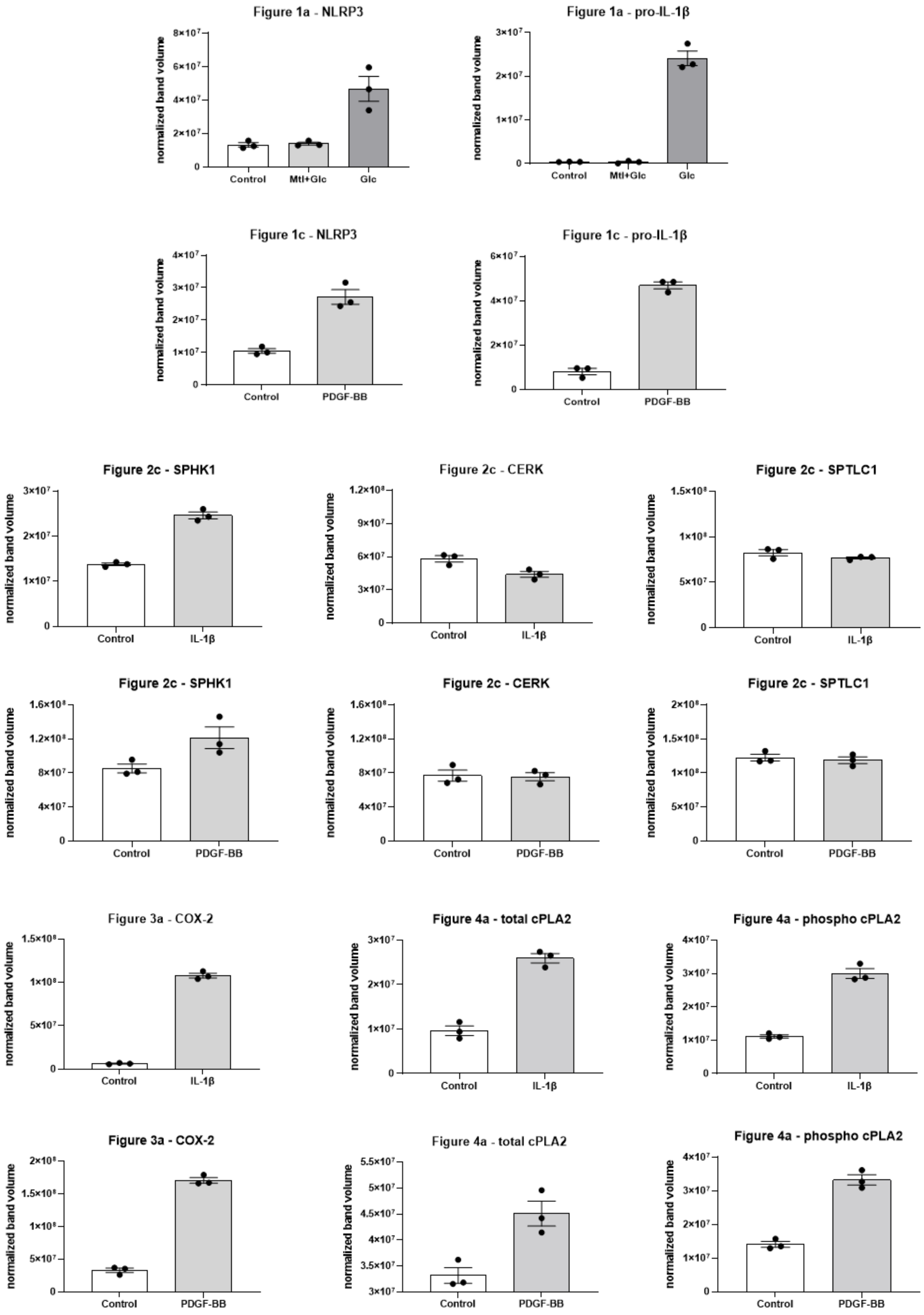

Supplementary Figure S5. Protein band quantification for each of the blots presented in Figures 1a, 1c, 2c 3a and 4a. The normalization is obtained through the stain free total lane method. Each single point in the scatter plot represents a quantified band; Average band volumes and relative SEM are reported.

Supplementary Figure S6.

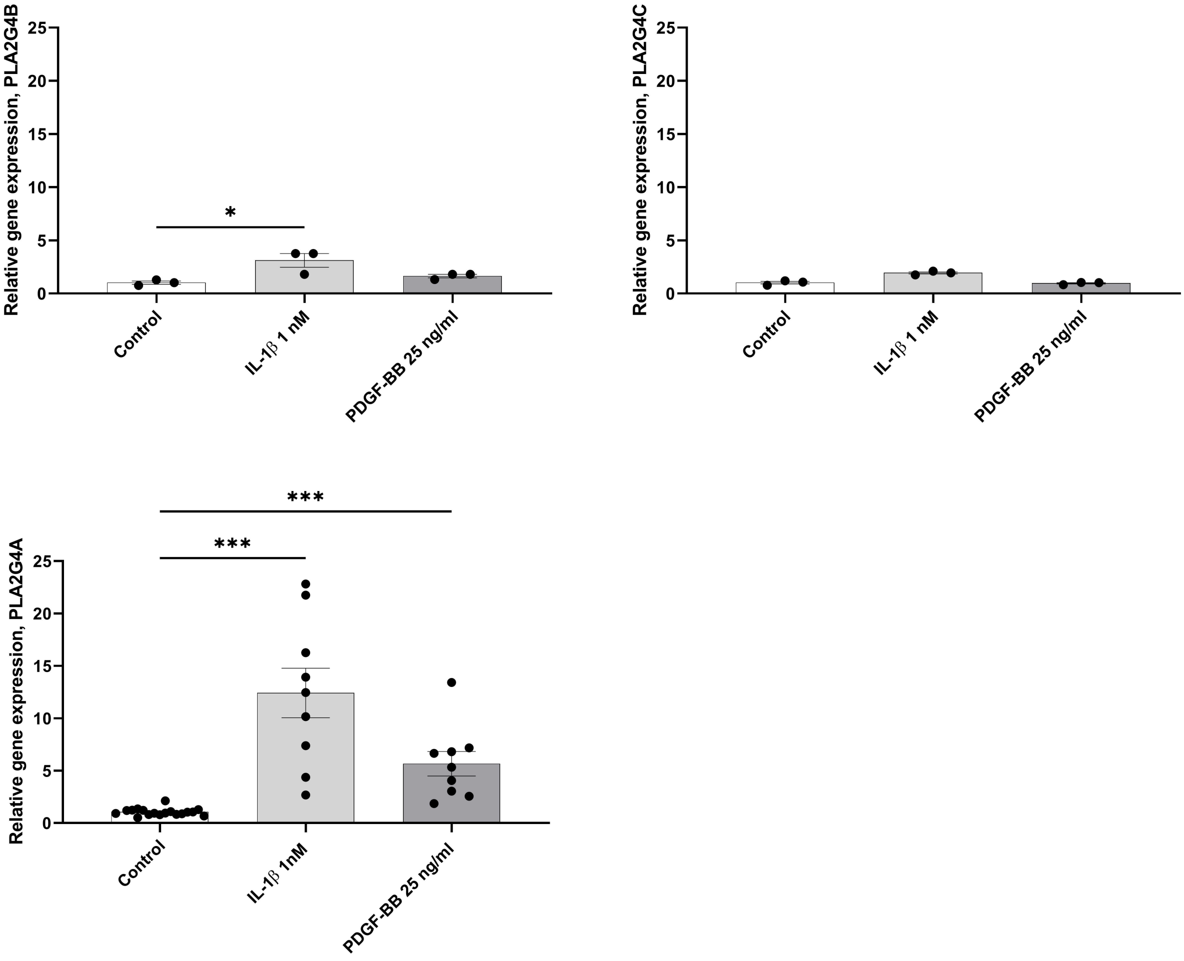

Supplementary Figure S6. Gene expression analysis of two other PLA2 present in human mesangial cells, PLA2G4B (cPLA2 group IVB) and PLA2G4C (iPLA2). The two selected PLA2 are upregulated by 24 h treatment with IL-1β. However, the regulation is not comparable with the levels reached by PLA2G4A (cPLA2 group IVA). * *P*<0.05; *** *P*<0.001. Data are reported as mean ± SEM. The same y axis range was used to emphasize the stronger upregulation of PLA2G4A. Data for PLA2G4 are the same as in Figure 4, C-D.

Supplementary Table S1. Lipidomic analysis. Results for lipid classes analysis.

CLASSES	Controls		IL-1β		PDGF-BB		Kruskal-Wallis *t*	Kruskal-Wallis *p* value	Multiple Comparisons IL-1β - ctrl	Multiple Comparisons PDGF-BB - control
	AVERAGE	SEM	AVERAGE	SEM	AVERAGE	SEM				
Cholestheryl Esthers (CE)	3.2564	0.1791	3.0964	0.2079	2.2544	0.2182	6.86	0.0236 *		q 0.0140 - p 0.0133 * (PDGF-BB<ctrl)
Diacylglycerols (DG)	1.5196	0.0708	1.5426	0.1089	1.1216	0.0551	7.94	0.0111 *		q 0.0170 - p 0.0162 * (PDGF-BB<ctrl)
Triacylglycerols (TG)	1.4642	0.0521	1.0450	0.1065	1.2334	0.2615	5.04	0.0746		
Phosphatidylcholines (PC)	58.9156	4.0153	60.8868	4.6674	67.7454	11.1605	0.06	0.9826		
Phosphoethanolamines (PE)	17.4214	1.1167	19.7104	1.4353	18.0674	2.1779	1.58	0.4815		
Lyso-Phosphatidylcholines (LPC)	0.4724	0.0396	0.4002	0.0343	0.3566	0.0570	2.94	0.2516		
Sphingomyelins (SM)	15.6650	0.8385	14.5832	0.7767	12.2114	0.5718	6.50	0.0312 *		q 0.0140 - p 0.0133 * (PDGF-BB<ctrl)
Ceramides [a] (Cer)	0.2280	0.0120	0.2126	0.0128	0.1794	0.0108	6.33	0.0331 *		q 0.0169 - p 0.0161 * (PDGF-BB<ctrl)
Dihydro-ceramides [a] (dhCer)	0.0098	0.0005	0.0095	0.0008	0.0079	0.0006	3.95	0.1427		
Glucosylceramides (GluCer)	0.2410	0.0141	0.2276	0.0103	0.1542	0.0061	9.64	0.0017 **		q 0.0039 - p 0.0037 ** (PDGF-BB<ctrl)
Lactosylceramides (LacCer)	0.1350	0.0068	0.1266	0.0061	0.1006	0.0074	6.63	0.0278 *		q 0.0125 - p 0.0119 * (PDGF-BB<ctrl)
Total sphingoid bases	0.1868	0.0124	0.1194	0.0087	0.0796	0.0068	12.04	<0.0001 ***	q 0.0345 - p 0.0658 ** (IL-1β<ctrl)	q 0.0006 - p 0.0005 *** (PDGF-BB<ctrl)
Total Ceramides [b]	0.6136	0.0304	0.5760	0.0288	0.4418	0.0172	8.82	0.0047 **		q 0.0061 - p 0.0058 ** (PDGF-BB<ctrl)
Total Sphingolipids [c]	16.4650	0.8757	15.2790	0.8127	12.7330	0.5878	6.50	0.0312 *		q 0.0140 - p 0,0133 * (PDGF-BB<ctrl)
Reserve lipids (TG, DG, CE)	6.2398	0.2474	5.6840	0.3363	4.6096	0.1901	9.38	0.0029 **		q 0.0025 - p 0.0024 ** (PDGF-BB<ctrl)
Total membrane lipids [d]	92.6154	5.7320	95.7566	6.6919	98.4658	13.0167	0.08	0.9680		
Total Lipids	99.5146	5.9712	101.9600	7.0520	103.5116	13.1709	0.14	0.9540		

[a] not glycosylated; [b] Cer, dhCer, GluCer, LacCer; [c] Cer, dhCer, GluCer, LacCer, SM,SG, dhSG, S1P, dhS1P, PhytoSG; [d] Cer, dhCer, GluCer, LacCer, SM, PC, PE. Average values represent nmol/cell count normalization factor. p, p value; q, q value; * P<0.05, ** P<0.01, *** P<0.001.

Supplementary Table S2. Lipidomic analysis. Results for each lipid species measured.

SPECIES	Controls		IL-1β		PDGF-BB		Kruskal-Wallis *t*	Kruskal-Wallis *p* value	Multiple Comparisons IL-1β - ctrl	Multiple Comparisons PDGF-BB - control
	AVERAGE	SEM	AVERAGE	SEM	AVERAGE	SEM				
CE 14:0	0.08481	0.00845	0.06811	0.00697	0.04576	0.00709	9.68	0.0014 **		q 0.0020 - p 0.0019 ** (PDGF-BB<ctrl)
CE 16:0	0.62012	0.04314	0.62596	0.03718	0.45656	0.03144	8.06	0.0092 **		q 0.0248 - q 0.0237 * (PDGF-BB<ctrl)
CE 16:1	0.30011	0.02341	0.29880	0.01655	0.23829	0.01310	6.62	0.0285 *		
CE 18:0	0.14221	0.00671	0.13627	0.01381	0.09680	0.01383	5.82	0.0478 *		q 0.0206 - p 0.0196 * (PDGF-BB<ctrl)
CE 18:1	1.13455	0.07969	1.14213	0.07346	0.83984	0.09711	5.46	0.0601		
CE 18:2	0.22296	0.01282	0.22774	0.01242	0.16849	0.02095	5.46	0.0601		
CE 20:3	0.08135	0.00494	0.06430	0.00507	0.05299	0.00399	8.88	0.0042 **	q 0.0471 - p 0.0897 * (IL-1β<ctrl)	q 0.0031 - p 0.0030 ** (PDGF-BB<ctrl)
CE 20:4	0.21936	0.01244	0.20026	0.01423	0.13578	0.02179	6.02	0.0431 *		q 0.0170 - p 0.0162 * (PDGF-BB<ctrl)
CE 20:5	0.15131	0.01682	0.12734	0.01121	0.09070	0.00602	7.98	0.0105 *		q 0.0061 - p 0.0058 ** (PDGF-BB<ctrl)
CE 22:5	0.12772	0.01865	0.07136	0.00816	0.05132	0.00757	9.29	0.0016 **	q 0.0433 - p 0.0413 * (IL-1β<ctrl)	q 0.0065 - p 0.0031 ** (PDGF-BB<ctrl)
CE 22:6	0.17189	0.01198	0.13428	0.02416	0.08816	0.00890	8.42	0.0068 **		q 0.0039 - p 0.0037 ** (PDGF-BB<ctrl)
DG 14:0/16:0	0.02892	0.00158	0.04763	0.01731	0.02143	0.00095	9.14	0.0035 **		
DG 16:0/16:0	0.06935	0.00165	0.12133	0.02664	0.06368	0.00308	10.82	0.0002 ***		
DG 14:0/18:1	0.02549	0.00221	0.01900	0.00196	0.01585	0.00111	8.06	0.0092 **		q 0.0049 - p 0.00047 ** (PDGF-BB<ctrl)
DG 16:0/16:1	0.08857	0.00603	0.08464	0.00512	0.08450	0.00534	0.86	0.6808		
DG 16:1/16:1	0.01609	0.00196	0.01366	0.00109	0.01835	0.00151	4.34	0.1182		
DG 16:0/18:0	0.03577	0.00078	0.06851	0.02607	0.03968	0.00170	7.62	0.0130 *		
DG 16:0/18:1	0.24270	0.01340	0.24376	0.01385	0.19060	0.01105	6.62	0.0285 *		
DG 16:1/18:0	0.03638	0.00202	0.03637	0.00181	0.04052	0.00206	2.94	0.2516		
DG 16:0/18:2	0.01959	0.00133	0.02040	0.00229	0.01826	0.00123	0.78	0.7027		
DG 18:0/18:1	0.17325	0.00903	0.15916	0.00912	0.14046	0.00601	5.46	0.0601		q 0.0206 - p 0.0196 * (PDGF-BB<ctrl)
DG 18:0/18:2	0.01601	0.00089	0.01746	0.00130	0.01491	0.00086	2.22	0.3532		
DG 18:1/18:1	0.13744	0.00749	0.12390	0.00866	0.10392	0.00701	5.84	0.0458 *		q 0.0170 - p 0.0162 * (PDGF-BB<ctrl)
DG 16:0/20:3	0.01165	0.00077	0.01006	0.00059	0.01198	0.00083	3.02	0.2311		
DG 16:0/20:4	0.01898	0.00087	0.02069	0.00115	0.01227	0.00041	9.98	0.0009 ***		q 0.0248 - p 0.0237 * (PDGF-BB<ctrl)
DG 18:0/20:3	0.12251	0.00711	0.13140	0.00868	0.12778	0.00961	0.32	0.8710		
DG 18:0/20:4	0.37040	0.01282	0.31079	0.01156	0.14920	0.00379	12.5	<0.0001 ***	q 0.0405 - p 0.0711 * (IL-1β<ctrl)	q 0.0004 - p 0.0004 *** (PDGF-BB<ctrl)
DG 18:1/20:3	0.02022	0.00097	0.02568	0.00132	0.02547	0.00167	6.02	0.0431 *	q 0.0423 - p 0.0284 * (IL-1β>ctrl)	q 0.0423- p 0.0403 * (PDGF-BB>ctrl)
DG 18:1/20:4	0.04776	0.00216	0.05475	0.00162	0.02738	0.00086	11.18	0.0001 ***		
DG 16:0/22:6	0.02475	0.00808	0.02158	0.00900	0.00825	0.00056	9.92	0.0010 ***		q 0.0020 - p 0.0019 **(PDGF-BB<ctrl)
DG 18:1/22:6	0.01382	0.00092	0.01188	0.00088	0.00718	0.00073	9.36	0.0031 **		q 0.0031 - p 0.0030 ** (PDGF-BB<ctrl)
TG 48:0	0.09285	0.02098	0.07026	0.01085	0.05030	0.00722	2.78	0.2648		
TG 48:1	0.12594	0.01457	0.09098	0.00550	0.10755	0.01659	3.98	0.1373		
TG 48:2	0.04011	0.00256	0.02540	0.00192	0.03670	0.00641	5.84	0.0458 *	q 0.0170 - p 0.0162 * (IL-1β<ctrl)	
TG 50:0	0.05039	0.00614	0.04865	0.00772	0.03879	0.00321	2.34	0.3304		
TG 50:1	0.19829	0.00775	0.16617	0.01153	0.17304	0.02550	3.12	0.2231		
TG 50:2	0.15728	0.00234	0.11400	0.01066	0.14475	0.02503	5.46	0.0601	q 0.0206 - p 0.0196 * (IL-1β<ctrl)	
TG 50:3	0.03444	0.00171	0.02316	0.00233	0.03162	0.00524	6.14	0.0380 *	q 0.0140 - p 0.0133 * (IL-1β<ctrl)	
TG 52:0	0.00984	0.00062	0.00945	0.00136	0.00996	0.00097	0.38	0.8580		
TG 52:1	0.09807	0.00615	0.08212	0.00802	0.10813	0.02796	1.62	0.4661		
TG 52:2	0.23020	0.00480	0.17190	0.02063	0.20958	0.05982	3.38	0.2009		
TG 52:3	0.11373	0.00417	0.07166	0.00929	0.10695	0.02838	6.86	0.0236 *	q 0.0093 - p 0.0089 ** (IL-1β<ctrl)	
TG 54:2	0.08667	0.00381	0.05733	0.00743	0.06791	0.01692	4.88	0.0837		
TG 54:3	0.09396	0.00524	0.04983	0.00652	0.06691	0.01663	6.72	0.0259 *	q 0.0115 - p 0.0109 * (IL-1β<ctrl)	q 0.0471 - p 0.0897 * (PDGF-BB<ctrl)
TG 54:4	0.01850	0.00171	0.01159	0.00209	0.01404	0.00348	4.02	0.1322		
TG 54:5	0.03474	0.00662	0.01656	0.00149	0.01764	0.00594	6.02	0.0431 *	q 0.0423 -p 0.0403 * (IL-1β<ctrl)	q 0.0423 - p 0.0284 * (PDGF-BB<ctrl)
TG 56:5	0.01465	0.00308	0.00733	0.00036	0.01280	0.00534	1.82	0.4441		
TG 56:6	0.03593	0.00290	0.01483	0.00170	0.02067	0.00915	6.00	0.0440 *	q 0.0356 - p 0.0339 * (IL-1β<ctrl)	q 0.0356 - p 0.0339 * (PDGF-BB<ctrl)
TG 56:7	0.02841	0.00169	0.01371	0.00115	0.01629	0.00611	6.00	0.0440 *	q 0.0356 - p 0.0339 * (IL-1β<ctrl)	q 0.0356 - p 0.0339 * (PDGF-BB<ctrl)
PC 30:0	1.63786	0.12638	2.49021	0.32908	2.06330	0.52141	5.04	0.0746		

SPECIES	Controls		IL-1β		PDGF-BB		Kruskal-Wallis *t*	Kruskal-Wallis *p* value	Multiple Comparisons IL-1β - ctrl	Multiple Comparisons PDGF-BB - control
	AVERAGE	SEM	AVERAGE	SEM	AVERAGE	SEM				
PC 32:0	4.19725	0.30603	4.64546	0.28960	4.55278	1.17269	1.82	0.4441		
PC 32:1	6.38078	0.46993	7.08387	0.51059	9.57318	2.38626	1.62	0.4661		
PC 32:2	1.47161	0.12237	1.60118	0.12608	2.22702	0.59223	0.98	0.6500		
PC 32:3	0.22881	0.01976	0.24573	0.01906	0.30106	0.08815	0.42	0.8322		
PC 34:1	15.15711	1.05827	15.24262	1.04559	17.52186	2.60822	0.38	0.858		
PC 34:2	4.57917	0.35250	4.69568	0.36577	6.13700	0.98684	1.82	0.4441		
PC 34:3	0.88193	0.07039	0.88032	0.08824	1.05598	0.18746	0.56	0.7825		
PC 34:4	0.23282	0.02226	0.23961	0.02117	0.28865	0.07012	0.02	0.9976		
PC 36:1	4.13982	0.27775	4.14860	0.27069	3.67098	0.38457	0.74	0.7249		
PC 36:2	6.64864	0.43391	6.59276	0.56262	7.74356	0.85845	1.04	0.6200		
PC 36:3	1.03899	0.07298	1.09965	0.09394	1.60976	0.33918	3.02	0.2311		
PC 36:4	1.80872	0.12037	1.97349	0.13459	1.81534	0.20743	0.42	0.8322		
PC 36:5	0.63431	0.04609	0.66742	0.06120	0.60666	0.07975	0.38	0.8580		
PC 38:2	0.14023	0.03215	0.10947	0.01016	0.15077	0.02787	2.22	0.3532		
PC 38:3	0.68202	0.05809	0.66449	0.06339	0.71622	0.06783	0.26	0.8980		
PC 38:4	3.03445	0.32288	2.85093	0.41081	2.63226	0.45765	0.26	0.0898		
PC 38:5	2.79206	0.17721	2.52915	0.20124	2.19123	0.21312	2.66	0.2839		
PC 38:6	0.91089	0.06210	0.94309	0.08137	0.83622	0.08366	0.74	0.7249		
PC 40:4	0.20049	0.02409	0.19180	0.01258	0.19643	0.02407	0.18	0.9254		
PC 40:5	0.74630	0.10865	0.53434	0.03259	0.42579	0.04457	8.42	0.0068 **		q 0.0390 - p 0.0370 ** (PDGF-BB<ctrl)
PC 40:6	0.87813	0.04904	0.93662	0.07078	0.88911	0.08860	0.26	0.8980		
PC 40:7	0.41383	0.02335	0.44128	0.03234	0.45680	0.04773	0.38	0.8580		
PC 40:8	0.07929	0.00819	0.07910	0.00364	0.08328	0.01105	0.14	0.9537		
LPC 16:0	0.14966	0.02012	0.12722	0.00899	0.09944	0.01528	3.44	0.1837		
LPC 16:1	0.02789	0.00316	0.03530	0.00454	0.04049	0.00503	3.78	0.1587		
LPC 18:0	0.18330	0.01464	0.10562	0.00951	0.07228	0.01463	9.98	0.0009 ***	q 0.0248 - p 0.0237 * (IL-1β<ctrl)	q 0.0050 - p 0.0024 ** (PDGF-BB<ctrl)
LPC 18:1	0.09364	0.00700	0.11415	0.01396	0.12745	0.02155	1.09	0.4027		
LPC 20:4	0.01806	0.00101	0.01777	0.00217	0.01711	0.00390	1.68	0.4585		
PE 34:1	1.31058	0.07924	1.50964	0.10490	1.85118	0.30525	2.96	0.2391		
PE 34:2	0.41881	0.03226	0.51911	0.03557	0.66618	0.11755	4.02	0.1322		
PE 34:3	0.06933	0.00482	0.08125	0.00872	0.09084	0.01822	1.14	0.6010		
PE 34:4	0.02077	0.00240	0.02296	0.00221	0.02279	0.00334	0.24	0.9113		
PE 36:1	2.69397	0.14110	2.98553	0.20860	2.75051	0.24673	2.24	0.3416		
PE 36:2	2.03162	0.12194	2.42320	0.20869	2.07713	0.17405	2.94	0.2516		
PE 36:3	0.25213	0.01899	0.28741	0.02308	0.27683	0.01804	1.86	0.4163		
PE 36:4	0.28274	0.01926	0.37282	0.02352	0.34578	0.02854	6.32	0.0333 *	q 0.0170 - p 0.0162 * (IL-1β<ctrl)	q 0.0346 - p 0.0660 * (PDGF-BB<ctrl)
PE 36:5	0.11513	0.00601	0.14081	0.00911	0.11510	0.01057	4.38	0.1097		
PE 36:6	0.02659	0.00190	0.03312	0.00456	0.02244	0.00194	4.22	0.1226		
PE 38:2	0.18760	0.01538	0.22382	0.02139	0.22131	0.04083	1.82	0.4441		
PE 38:3	0.68573	0.05982	0.83041	0.07766	0.85053	0.18817	2.06	0.3833		
PE 38:4	3.34421	0.19452	3.71081	0.26010	3.59071	0.67493	1.26	0.5642		
PE 38:5	1.34979	0.08276	1.66880	0.13705	1.38935	0.21218	3.42	0.1905		
PE 38:6	0.74940	0.05153	0.87253	0.07592	0.59519	0.08742	3.92	0.1449		
PE 38:7	0.11494	0.00853	0.12284	0.01281	0.09949	0.01175	2.66	0.2839		
PE 38:8	0.05810	0.00462	0.06331	0.00518	0.05903	0.00667	1.04	0.6200		
PE 40:4	0.43283	0.02924	0.46443	0.01653	0.36143	0.04450	3.38	0.2009		
PE 40:5	0.99095	0.07429	1.03920	0.04604	0.82274	0.12141	2.78	0.2648		
PE 40:6	1.38795	0.10798	1.42327	0.09456	1.17831	0.16888	2.54	0.3040		
PE 40:7	0.81618	0.07876	0.83672	0.07551	0.60881	0.08175	4.88	0.0837		
PE 40:8	0.08205	0.01112	0.07829	0.00594	0.07159	0.00856	0.56	0.7825		
SM 14:0	0.50672	0.03157	0.51563	0.03131	0.42027	0.02386	4.34	0.1182		
SM 15:0	0.34367	0.01758	0.32424	0.01865	0.22838	0.00802	9.42	0.0024 **		q 0.0061 - p 0.0058 ** (PDGF-BB<ctrl)

SPECIES	Controls		IL-1β		PDGF-BB		Kruskal-Wallis *t*	Kruskal-Wallis *p* value	Multiple Comparisons IL-1β - ctrl	Multiple Comparisons PDGF-BB - control
	AVERAGE	SEM	AVERAGE	SEM	AVERAGE	SEM				
SM 16:0	7.41404	0.41121	6.93641	0.35208	5.72897	0.27538	6.50	0.0312 *		q 0.0140 - p 0.0133 * (PDGF-BB<ctrl)
dhSM 16:0	0.36600	0.01178	0.45877	0.04133	0.48930	0.03135	5.82	0.0478 *	q 0.0471 - p 0.0897 * (IL-1β>ctrl)	q 0.0206 - p 0.0196 * (PDGF-BB>ctrl)
SM 16:1	0.66278	0.03757	0.65056	0.03317	0.50428	0.02060	8.06	0.0092 **		q 0.0093 - p 0.0089 ** (PDGF-BB<ctrl)
SM 17:0	0.36953	0.02076	0.34269	0.01366	0.23607	0.00894	9.78	0.0012 **		p 0.0031 - p 0.0030 ** (PDGF-BB<ctrl)
dhSM 17:0	0.10592	0.00680	0.10767	0.00280	0.08070	0.00392	8.18	0.0081 **		
SM 17:1	0.04741	0.00241	0.04268	0.00163	0.02923	0.00169	10.22	0.0006 ***		q 0.0020 - p 0.0019 ** (PDGF-BB<ctrl)
SM 18:0	0.24536	0.01244	0.23337	0.01382	0.20656	0.00737	5.36	0.0650		q 0.0248 - p 0.0237 * (PDGF-BB<ctrl)
SM 18:1	0.07503	0.00564	0.06423	0.00459	0.05259	0.00238	6.26	0.0348 *		q 0.0140 - p 0.0133 * (PDGF-BB<ctrl)
SM 20:0	0.06251	0.00655	0.05340	0.00249	0.05292	0.00502	1.22	0.5824		
SM 22:0	0.51974	0.04558	0.45211	0.02632	0.45289	0.01231	2.66	0.2839		
SM 22:1	0.23553	0.01372	0.21400	0.01234	0.17898	0.00939	7.22	0.0193 *		q 0.0093 - p 0.0089 ** (PDGF-BB<ctrl)
SM 23:0	0.14791	0.01073	0.13953	0.00715	0.10549	0.00592	7.98	0.0105 *		q 0.0115 - p 0.0109 * (PDGF-BB<ctrl)
SM 23:1	0.24266	0.01256	0.21260	0.00926	0.16778	0.00872	9.62	0.0018 **		q 0.0025 - p 0.0024 ** (PDGF-BB<ctrl)
SM 24:0	0.72975	0.03817	0.66968	0.04458	0.64108	0.03771	2.00	0.3896		
SM 24:1	2.72590	0.13511	2.41613	0.15041	2.02288	0.09500	7.28	0.0176 *		q 0.0076 - p 0.0072 ** (PDGF-BB<ctrl)
SM 24:2	0.72556	0.03366	0.62717	0.03828	0.49675	0.02361	8.88	0.0042 **		q 0.0031 - p 0.0030 ** (PDGF-BB<ctrl)
SM 25:0	0.04082	0.00334	0.03494	0.00268	0.03806	0.00388	2.06	0.3833		
SM 25:1	0.09823	0.00480	0.08748	0.00873	0.07822	0.00449	4.56	0.0995		
Cer 16:0	0.09945	0.00497	0.09921	0.00530	0.08400	0.00534	3.86	0.1503		
Cer 18:0	0.00352	0.00013	0.00288	0.00019	0.00273	0.00021	6.74	0.0248 *	q 0.0423 - p 0.0403 * (IL-1β<ctrl)	q 0.0340 - p 0.0162 * (PDGF-BB<ctrl)
Cer 20:0	0.00098	0.00006	0.00110	0.00008	0.00073	0.00004	10.22	0.0006 ***		
Cer 22:0	0.00895	0.00044	0.00782	0.00043	0.00909	0.00046	3.86	0.1503		
Cer 24:0	0.04993	0.00361	0.05123	0.00386	0.04554	0.00272	1.82	0.4441		
Cer 24:1	0.06510	0.00386	0.05032	0.00331	0.03718	0.00250	11.06	0.0001 ***		q 0.0009 - p 0.0009 *** (PDGF-BB<ctrl)
dhCer 16:0	0.00523	0.00030	0.00514	0.00046	0.00448	0.00044	2.18	0.3651		
dhCer 18:0	0.00066	0.00008	0.00037	0.00005	0.00045	0.00005	6.50	0.0312 *	q 0.0140 - p 0.0133 * (IL-1β<ctrl)	q 0.0405 - p 0.0771 * (PDGF-BB<ctrl)
dhCer 22:0	0.00078	0.00004	0.00077	0.00008	0.00077	0.00005	0.50	0.8068		
dhCer 24:0	0.00140	0.00006	0.00182	0.00014	0.00126	0.00006	8.88	0.0420 **		
dhCer 24:1	0.00171	0.00010	0.00136	0.00014	0.00095	0.00007	9.74	0.0014 **		q 0.0020 p 0.0019 ** (PDGF-BB<ctrl)
GluCer 16:0	0.08086	0.00437	0.08494	0.00431	0.04768	0.00260	9.50	0.0021 **		q 0.0140 - p 0.0133 * (PDGF-BB<ctrl)
GluCer 18:0	0.00461	0.00017	0.00416	0.00028	0.00341	0.00020	7.91	0.0104 *		q 0.0540 - p 0.0052 ** (PDGF-BB<ctrl)
GluCer 20:0	0.00075	0.00005	0.00078	0.00005	0.00075	0.00004	0.26	0.8893		
GluCer 22:0	0.03008	0.00189	0.02697	0.00143	0.02498	0.00103	4.56	0.0995		
GluCer 24:0	0.04929	0.00356	0.04647	0.00137	0.03801	0.00134	6.86	0.0236		q 0.0140 - p 0.0133 * (PDGF-BB<ctrl)
GluCer 24:1	0.07532	0.00463	0.06421	0.00316	0.03942	0.00153	11.18	0.0001 ***		q 0.0009 - p 0.0009 *** (PDGF-BB<ctrl)
LacCer 16:0	0.01916	0.00082	0.01807	0.00096	0.01261	0.00050	9.78	0.0012 **		q 0.0031 - p 0.0030 ** (PDGF-BB<ctrl)
LacCer 18:0	0.00140	0.00007	0.00142	0.00008	0.00118	0.00009	3.86	0.1503		
LacCer 20:0	0.00021	0.00003	0.00021	0.00002	0.00017	0.00001	1.90	0.4096		
LacCer 22:0	0.02037	0.00093	0.01988	0.00126	0.01615	0.00042	7.44	0.0152 *		q 0.0115 - p 0.0109 * (PDGF-BB< ctrl)
LacCer 24:0	0.02702	0.00072	0.02826	0.00144	0.02241	0.00204	4.46	0.1055		
LacCer 24:1	0.06666	0.00456	0.05864	0.00315	0.04787	0.00549	4.22	0.1226		
SG	0.17169	0.01206	0.10887	0.00755	0.07084	0.00618	12.02	<0.0001 ***	q 0.0346 - p 0.0660 * (IL-1β<ctrl)	q 0.0006 - p 0.0005 *** (PDGF-BB<ctrl)
dhSG	0.01274	0.00052	0.00848	0.00096	0.00734	0.00044	9.14	0.0035 **	q 0.0298 - p 0.0284 * (IL-1β<ctrl)	q 0.0079- p 0.0037 ** (PDGF-BB<ctrl)
PhytoSG	0.00154	0.00010	0.00122	0.00019	0.00058	0.00006	10.22	0.0006 ***		q 0.0020 - p 0.0019 ** (PDGF-BB<ctrl)
S1P	0.00066	0.00003	0.00066	0.00007	0.00071	0.00010	0.14	0.9537		
dhS1P	0.00005	0.00001	0.00005	0.00001	0.00009	0.00001	6.63	0.0275 *		q 0.0247 - p 0.0235 * (PDGF-BB>ctrl)

Average values represent nmol/cell count normalization factor. p, p value; q, q value; * *P* <0.05, ** *P* <0.01, *** *P* <0.001.

Supplementary Table S3. Lipidomic data. Results for each lipid class measured, normalized nmol amounts.

Lipid Group		Lipid Class	control #1	control #2	control #3	control #4	control #5	IL-1β #1	IL-1β #2	IL-1β #3	IL-1β #4	IL-1β #5	PDGF-BB #1	PDGF-BB #2	PDGF-BB #3	PDGF-BB #4	PDGF-BB #5
Cholesteryl esters and Acylglycerols		**CE**	2.9948	3.3708	2.7631	3.8133	3.3399	3.8705	2.8542	2.7860	2.7783	3.1938	2.2047	2.4079	2.1644	2.9246	1.5704
		**DG**	1.2915	1.6214	1.4203	1.6646	1.6005	1.6255	1.5620	1.2144	1.8750	1.4364	1.2945	1.0982	1.0836	0.9594	1.1727
		**TG**	1.6150	1.4257	1.3868	1.5546	1.3379	1.4203	1.1311	0.8559	0.8527	0.9646	0.7204	0.9022	1.0484	1.2851	2.2120
Phospholipids/phosphatides		**PC**	47.3528	56.7577	54.5512	68.8331	67.0828	78.4285	61.6047	52.3744	54.0758	57.9509	46.9975	48.6572	73.9713	61.1757	107.9245
		**PE**	15.3294	15.8742	15.7466	20.8672	19.2897	24.7270	20.6987	17.6258	16.5203	18.9798	13.8297	14.9810	15.5367	20.5015	25.4874
		**LPC**	0.3684	0.5427	0.3984	0.4813	0.5720	0.5199	0.4109	0.3271	0.3388	0.4038	0.2173	0.2796	0.3860	0.3474	0.5535
Sphingolipids	Ceramides	**Cer**	0.1973	0.2219	0.2225	0.2709	0.2270	0.2336	0.2458	0.1742	0.1991	0.2101	0.1912	0.1905	0.1519	0.1557	0.2071
		**GluCer**	0.1919	0.2462	0.2395	0.2802	0.2467	0.2417	0.2526	0.1953	0.2150	0.2331	0.1534	0.1721	0.1564	0.1338	0.1556
		**LacCer**	0.1170	0.1458	0.1448	0.1466	0.1199	0.1426	0.1330	0.1067	0.1302	0.1199	0.0920	0.0915	0.0827	0.1148	0.1209
		**dhCer**	0.0094	0.0108	0.0108	0.0095	0.0083	0.0108	0.0113	0.0071	0.0081	0.0101	0.0069	0.0084	0.0062	0.0089	0.0091
		**Total ceramides**	0.5156	0.6248	0.6177	0.7072	0.6019	0.6288	0.6426	0.4833	0.5524	0.5732	0.4434	0.4626	0.3972	0.4132	0.4926
	Sphingoid bases	**SB + PSB**	0.1519	0.1860	0.1685	0.2220	0.2050	0.1397	0.1398	0.0978	0.1067	0.1124	0.0827	0.1016	0.0755	0.0605	0.0774
	Sphingomyelins	**SM**	13.2082	17.2800	14.5519	17.7069	15.5783	16.0866	15.9912	11.9273	13.8501	15.0614	12.5510	13.6728	12.1239	10.1689	12.5402
**TOTAL sphingolipids**			13.8758	18.0908	15.3381	18.6361	16.3852	16.8551	16.7737	12.5083	14.5091	15.7469	13.0772	14.2370	12.5967	10.6426	13.1103
**TOTAL lipids**			82.8277	97.6833	91.6043	115.8501	109.6080	127.4467	105.0352	87.6919	90.9499	98.6762	78.3414	82.5632	106.7871	97.8363	152.0308

Supplementary Table S4. Lipidomic data. Results for each lipid species measured, normalized nmol amounts.

Lipid Species	control #1	control #2	control #3	control #4	control #5	IL-1β #1	IL-1β #2	IL-1β #3	IL-1β #4	IL-1β #5	PDGF-BB #1	PDGF-BB #2	PDGF-BB #3	PDGF-BB #4	PDGF-BB #5
CE 14:0	0.101206	0.074001	0.070492	0.109145	0.069199	0.095652	0.059899	0.059895	0.059590	0.065537	0.043631	0.065050	0.039364	0.056658	0.024080
CE 16:0	0.529504	0.653491	0.514615	0.743467	0.659530	0.758044	0.552582	0.588287	0.575598	0.655295	0.424760	0.467216	0.496769	0.538791	0.355246
CE 16:1	0.269607	0.295948	0.237854	0.375426	0.321708	0.351980	0.267292	0.268196	0.284807	0.321740	0.213048	0.252445	0.257467	0.267372	0.201107
CE 18:0	0.127905	0.162078	0.129862	0.153510	0.137715	0.187193	0.116413	0.116497	0.116959	0.144280	0.093679	0.113751	0.078193	0.139000	0.059377
CE 18:1	0.962689	1.149799	0.959515	1.376591	1.224140	1.408826	1.021297	1.052072	1.035087	1.193369	0.801587	0.896142	0.779158	1.160636	0.561657
CE 18:2	0.190752	0.221573	0.198972	0.249037	0.254485	0.270447	0.203336	0.205436	0.220753	0.238715	0.160096	0.185009	0.170794	0.227973	0.098570
CE 20:3	0.067175	0.094336	0.077173	0.090593	0.077467	0.081256	0.065940	0.056685	0.051513	0.066107	0.055793	0.052130	0.039923	0.064795	0.052322
CE 20:4	0.197959	0.226008	0.186032	0.256206	0.230581	0.243901	0.192953	0.193213	0.157210	0.214025	0.137161	0.157274	0.119917	0.198426	0.066121
CE 20:5	0.204287	0.165147	0.103348	0.151367	0.132381	0.149826	0.152703	0.093349	0.127903	0.112904	0.113890	0.083035	0.081949	0.091091	0.083546
CE 22:5	0.196645	0.116772	0.118113	0.123813	0.083233	0.099681	0.077234	0.053999	0.057870	0.067996	0.059544	0.045786	0.032864	0.067081	*not detected*
CE 22:6	0.147111	0.211650	0.167090	0.184103	0.149476	0.223653	0.144533	0.098401	0.090966	0.113849	0.101511	0.090048	0.068000	0.112808	0.068415
TG 48:0	0.145439	0.050432	0.047074	0.083237	0.138090	0.105600	0.082816	0.054131	0.063947	0.044818	0.028483	0.052384	0.058183	0.041565	0.070868
TG 48:1	0.156607	0.103525	0.102636	0.166209	0.100714	0.109406	0.094314	0.077788	0.081973	0.091402	0.062526	0.090006	0.129897	0.097193	0.158145
TG 48:2	0.039117	0.038279	0.037654	0.050069	0.035454	0.031546	0.028000	0.021457	0.021987	0.023989	0.020849	0.031028	0.044396	0.029800	0.057447
TG 50:0	0.073058	0.041080	0.038338	0.047966	0.051493	0.075427	0.055644	0.036367	0.042683	0.033133	0.031704	0.039359	0.031363	0.044489	0.047052
TG 50:1	0.220275	0.193565	0.184356	0.212395	0.180874	0.205971	0.175524	0.143097	0.145560	0.160709	0.108289	0.145790	0.158284	0.193580	0.259246
TG 50:2	0.160936	0.160912	0.154761	0.160608	0.149187	0.152840	0.119402	0.098266	0.093335	0.106160	0.091339	0.111250	0.130699	0.154804	0.235633
TG 50:3	0.032058	0.030246	0.036296	0.040035	0.033586	0.030916	0.024811	0.021543	0.016770	0.021750	0.020032	0.025452	0.029016	0.032790	0.050817
TG 52:0	0.011327	0.011082	0.008237	0.009858	0.008688	0.013223	0.012178	0.007871	0.007559	0.006400	0.009630	0.009643	0.007176	0.010114	0.013229
TG 52:1	0.112966	0.106249	0.093657	0.100400	0.077053	0.110618	0.085424	0.069126	0.065037	0.080373	0.058173	0.072654	0.086666	0.108166	0.214988
TG 52:2	0.222508	0.233992	0.235628	0.242766	0.216086	0.243803	0.184898	0.137013	0.128062	0.165726	0.118237	0.134120	0.140681	0.215649	0.439231
TG 52:3	0.103619	0.113313	0.122417	0.123811	0.105467	0.103345	0.078920	0.053779	0.053180	0.069069	0.063429	0.067321	0.082012	0.105403	0.216597
TG 54:2	0.091085	0.093811	0.091309	0.084333	0.072787	0.083537	0.062476	0.044290	0.043050	0.053289	0.037710	0.042774	0.052226	0.076848	0.129993
TG 54:3	0.087253	0.093598	0.099338	0.110209	0.079419	0.070529	0.057592	0.038734	0.034534	0.047762	0.036637	0.041947	0.052111	0.076232	0.127631
TG 54:4	0.012845	0.018881	0.022793	0.020906	0.017074	0.018029	0.014700	0.007910	0.006952	0.010361	0.007254	0.009412	0.010316	0.016766	0.026435
TG 54:5	0.057661	0.039249	0.031347	0.026583	0.018868	0.018834	0.018040	0.013359	0.019911	0.012635	0.006444	0.009864	0.009154	0.025418	0.037318
TG 56:5	0.019857	0.021368	0.016645	0.010617	0.004766	0.008682	0.007311	0.007055	0.006493	0.007116	0.004135	0.005202	0.006289	0.015897	0.032455
TG 56:6	0.038453	0.042676	0.037085	0.036211	0.025231	0.020434	0.014477	0.012217	0.010685	0.016345	0.007829	0.008146	0.010311	0.021079	0.055960
TG 56:7	0.029966	0.033397	0.027220	0.028410	0.023057	0.017551	0.014539	0.011903	0.010968	0.013591	0.007738	0.005867	0.009652	0.019307	0.038910
DG 14:0/16:0	0.025040	0.028520	0.025981	0.032185	0.032866	0.033625	0.033934	0.023306	0.116451	0.030844	0.024239	0.021596	0.020822	0.018365	0.022152
DG 16:0/16:0	0.065762	0.071669	0.066387	0.074496	0.068460	0.097148	0.099975	0.076559	0.225954	0.106995	0.074043	0.065268	0.060629	0.055288	0.063193
DG 14:0/18:1	0.019147	0.026288	0.022065	0.028390	0.031565	0.021102	0.021250	0.012989	0.023676	0.015991	0.018590	0.014561	0.014777	0.012988	0.018325
DG 16:0/16:1	0.070035	0.093232	0.079754	0.096287	0.103536	0.093999	0.089350	0.064867	0.085578	0.089421	0.100780	0.085401	0.079367	0.068317	0.088651
DG 16:1/16:1	0.012924	0.015048	0.013267	0.015541	0.023674	0.015859	0.013201	0.010111	0.016042	0.013092	0.023101	0.018939	0.017386	0.013691	0.018610
DG 16:0/18:0	0.032907	0.035924	0.037350	0.036980	0.035674	0.046325	0.047119	0.037990	0.172512	0.038624	0.042429	0.042195	0.037029	0.034273	0.042456
DG 16:0/18:1	0.195989	0.261383	0.237086	0.274716	0.244350	0.270867	0.256241	0.190771	0.245547	0.255388	0.223009	0.184258	0.175241	0.162232	0.208263
DG 16:1/18:0	0.029986	0.039652	0.033639	0.040940	0.037701	0.039123	0.037351	0.030125	0.040347	0.034907	0.045261	0.044083	0.041132	0.033856	0.038273
DG 16:0/18:2	0.015091	0.021179	0.018292	0.020710	0.022662	0.020961	0.019307	0.013839	0.028148	0.019767	0.021693	0.017327	0.017694	0.014514	0.020096
DG 18:0/18:1	0.144120	0.186667	0.166710	0.196900	0.171859	0.182816	0.171128	0.128466	0.155126	0.158256	0.159645	0.139798	0.134599	0.123193	0.145088
DG 18:0/18:2	0.012893	0.017564	0.015116	0.017275	0.017219	0.019082	0.017551	0.013766	0.021215	0.015681	0.017580	0.014779	0.015416	0.012212	0.014560
DG 18:1/18:1	0.113297	0.152547	0.126780	0.148473	0.146103	0.144809	0.134590	0.095391	0.130462	0.114272	0.123343	0.093908	0.099785	0.085903	0.116678
DG 16:0/20:3	0.009125	0.013454	0.010858	0.012793	0.012033	0.011082	0.010071	0.008089	0.011438	0.009635	0.014536	0.011324	0.012152	0.009418	0.012447
DG 16:0/20:4	0.016626	0.021601	0.018125	0.020184	0.018341	0.022178	0.019513	0.017367	0.024083	0.020315	0.012905	0.012192	0.010893	0.012117	0.013256
DG 18:0/20:3	0.099312	0.128174	0.113957	0.139267	0.131858	0.156341	0.144106	0.106843	0.120635	0.129086	0.156926	0.121513	0.137761	0.098730	0.123955
DG 18:0/20:4	0.339965	0.395430	0.345671	0.403721	0.367200	0.337938	0.336362	0.294729	0.305670	0.279241	0.161893	0.149988	0.143108	0.139952	0.151078
DG 18:1/20:3	0.017463	0.022549	0.018458	0.021774	0.020840	0.028989	0.027911	0.021555	0.025684	0.024281	0.029785	0.023347	0.026723	0.020213	0.027276
DG 18:1/20:4	0.045566	0.054932	0.043325	0.050429	0.044536	0.056613	0.056835	0.048516	0.054594	0.057184	0.029364	0.025452	0.025161	0.028443	0.028482
DG 16:0/22:6	0.014502	0.018923	0.015063	0.018387	0.056871	0.014256	0.013716	0.009989	0.057464	0.012454	0.008227	0.006727	0.007892	0.008197	0.010210
DG 18:1/22:6	0.011718	0.016693	0.012374	0.015131	0.013181	0.012417	0.012506	0.009104	0.014387	0.010978	0.007141	0.005552	0.005984	0.007538	0.009695

Lipid Species	control #1	control #2	control #3	control #4	control #5	IL-1β #1	IL-1β #2	IL-1β #3	IL-1β #4	IL-1β #5	PDGF-BB #1	PDGF-BB #2	PDGF-BB #3	PDGF-BB #4	PDGF-BB #5
PC 30:0	1.330842	1.569867	1.492017	2.076649	1.719908	3.764437	2.179986	2.462156	1.962177	2.082273	1.365696	1.292633	1.978074	1.586505	4.093614
PC 32:0	3.150130	4.228220	3.999696	4.796998	4.811212	5.671292	4.680938	3.926155	4.319721	4.629170	3.036042	2.882464	4.308409	3.399134	9.137860
PC 32:1	4.868328	6.321086	6.027134	7.614119	7.073244	8.915793	7.137364	5.823844	6.581302	6.961069	6.210653	6.252175	9.314352	7.239889	18.848843
PC 32:2	1.117763	1.441774	1.318050	1.792571	1.687901	2.050210	1.639620	1.290718	1.475361	1.549981	1.405596	1.392732	2.186191	1.625597	4.524991
PC 32:3	0.173508	0.214423	0.209304	0.282927	0.263906	0.311503	0.258080	0.198119	0.225261	0.235672	0.175348	0.182182	0.292947	0.211207	0.643615
PC 34:1	11.913047	14.718874	14.345623	18.084937	16.723044	19.014845	15.617734	12.956623	13.793952	14.829939	12.455455	13.179289	19.183455	15.975304	26.815792
PC 34:2	3.485393	4.473594	4.271944	5.487944	5.176971	5.992080	4.862026	3.851588	4.208078	4.564644	4.333657	4.442299	6.869127	5.399601	9.640331
PC 34:3	0.665060	0.852797	0.820596	1.046616	1.024559	1.206403	0.903379	0.709711	0.747287	0.834795	0.722491	0.747868	1.162803	0.909679	1.737061
PC 34:4	0.178064	0.232020	0.189157	0.275798	0.289050	0.314288	0.242581	0.187519	0.213911	0.239739	0.171982	0.165286	0.285421	0.269022	0.551547
PC 36:1	3.445453	3.910935	3.756727	4.728386	4.857603	5.177557	4.129399	3.756069	3.664178	4.015783	2.689319	2.892745	4.195589	3.870100	4.707168
PC 36:2	5.416135	6.374568	6.215237	7.844145	7.393135	8.676840	6.786746	5.499103	5.838926	6.162165	5.740562	6.055979	9.228414	7.573311	10.119511
PC 36:3	0.810985	1.027998	0.966231	1.211012	1.178748	1.436698	1.152003	0.897562	0.976390	1.035578	1.022074	1.045239	1.765338	1.361097	2.855076
PC 36:4	1.462149	1.770444	1.659212	2.115245	2.036551	2.476506	2.002597	1.722392	1.778882	1.887073	1.276993	1.408618	2.125573	1.900859	2.364648
PC 36:5	0.522318	0.595263	0.571424	0.713041	0.769484	0.875044	0.733431	0.543501	0.571601	0.613533	0.415125	0.442224	0.727783	0.621026	0.827129
PC 38:2	0.064103	0.104530	0.239452	0.103669	0.189396	0.129593	0.136762	0.083200	0.098875	0.098918	0.117403	0.109129	0.166922	0.107052	0.253324
PC 38:3	0.487432	0.673033	0.657588	0.834455	0.757608	0.868733	0.746533	0.521763	0.567758	0.617679	0.564015	0.562820	0.879472	0.722019	0.852776
PC 38:4	3.115604	2.507665	2.393581	2.945235	4.210174	4.353263	2.419713	3.089956	2.103599	2.288143	1.466275	1.578989	3.388919	3.166315	3.560789
PC 38:5	2.283157	2.712483	2.586132	3.238196	3.140338	3.252092	2.662997	2.168956	2.190518	2.371173	1.637173	1.754616	2.513586	2.320954	2.729822
PC 38:6	0.736479	0.888284	0.828775	1.066214	1.034703	1.218826	1.029288	0.773641	0.817582	0.876099	0.636774	0.652347	0.985616	0.861733	1.044606
PC 40:4	0.128685	0.200957	0.211873	0.278047	0.182895	0.236083	0.201209	0.167688	0.169994	0.184002	0.130265	0.175691	0.244786	0.172716	0.258705
PC 40:5	0.856701	0.561020	0.518993	0.682661	1.112147	0.657556	0.541588	0.495719	0.476309	0.500543	0.308710	0.337975	0.474587	0.460576	0.547087
PC 40:6	0.744194	0.871920	0.811079	1.027307	0.936166	1.192401	0.973871	0.796163	0.828662	0.891982	0.685254	0.702394	1.029268	0.892736	1.135921
PC 40:7	0.344187	0.420447	0.386276	0.483074	0.435177	0.551768	0.477821	0.383249	0.392217	0.401342	0.358652	0.348336	0.544350	0.447766	0.584904
PC 40:8	0.053126	0.085546	0.075082	0.103809	0.078884	0.084665	0.089004	0.069000	0.073239	0.079600	0.071999	0.053204	0.120274	0.081482	0.089425
LPC 16:0	0.107459	0.207829	0.114925	0.131226	0.186866	0.158063	0.128650	0.106839	0.111852	0.130705	0.062492	0.076949	0.116421	0.092304	0.149049
LPC 16:1	0.021780	0.021034	0.025959	0.035986	0.034685	0.048205	0.039962	0.025486	0.024441	0.038412	0.026897	0.037684	0.049054	0.034183	0.054616
LPC 18:0	0.144576	0.212108	0.151362	0.198609	0.209852	0.140404	0.085828	0.098381	0.109665	0.093832	0.033920	0.048751	0.068148	0.099685	0.110874
LPC 18:1	0.079026	0.084670	0.089099	0.096089	0.119325	0.149296	0.136114	0.084181	0.079192	0.121983	0.083866	0.103262	0.137052	0.106427	0.206647
LPC 20:4	0.015522	0.017078	0.017068	0.019390	0.021256	0.023904	0.020330	0.012170	0.013620	0.018823	0.010170	0.012970	0.015364	0.014760	0.032306
PE 34:1	1.115082	1.273433	1.187651	1.550270	1.426447	1.889683	1.554080	1.321056	1.319926	1.463467	1.230556	1.272918	2.333726	1.637244	2.781462
PE 34:2	0.333738	0.385695	0.390646	0.514015	0.469967	0.644570	0.541372	0.444862	0.461651	0.503113	0.449495	0.437215	0.832790	0.570858	1.040549
PE 34:3	0.057695	0.066669	0.063549	0.085989	0.072767	0.111644	0.088514	0.065439	0.065115	0.075529	0.059471	0.059918	0.109746	0.071305	0.153741
PE 34:4	0.014006	0.018347	0.019017	0.026798	0.025705	0.030175	0.021859	0.022520	0.016374	0.023882	0.016458	0.016852	0.031104	0.018814	0.030701
PE 36:1	2.482750	2.487831	2.441374	3.135834	2.922046	3.735546	3.093890	2.701452	2.539640	2.857125	2.321338	2.505490	2.434865	3.683454	2.807418
PE 36:2	1.835083	1.872922	1.795932	2.368436	2.285750	3.172895	2.552503	2.078536	2.032067	2.280010	1.719005	1.863570	2.002987	2.730158	2.069951
PE 36:3	0.220506	0.236828	0.217764	0.320082	0.265455	0.372882	0.289125	0.262566	0.236392	0.276068	0.231287	0.251012	0.278659	0.337577	0.285596
PE 36:4	0.253380	0.270122	0.245262	0.352781	0.292151	0.463213	0.371132	0.334655	0.338659	0.356461	0.274773	0.299102	0.355233	0.439437	0.360352
PE 36:5	0.105556	0.105724	0.110450	0.137901	0.116012	0.168958	0.149345	0.130993	0.114402	0.140352	0.086604	0.094241	0.124705	0.142236	0.127690
PE 36:6	0.021733	0.024924	0.024198	0.030656	0.031441	0.048219	0.038738	0.024401	0.025112	0.029131	0.017700	0.019798	0.025295	0.028408	0.020996
PE 38:2	0.152397	0.177708	0.166152	0.239859	0.201902	0.290613	0.255644	0.180152	0.184893	0.207798	0.164511	0.185898	0.158906	0.217944	0.379291
PE 38:3	0.574534	0.572329	0.620477	0.836629	0.824687	1.085302	0.929073	0.679383	0.693149	0.765164	0.613592	0.650356	0.555969	0.857511	1.575215
PE 38:4	3.090990	2.968719	3.054297	3.954668	3.652384	4.657987	3.769155	3.437069	3.110627	3.579219	2.587323	2.922355	2.376807	4.028525	6.038531
PE 38:5	1.236242	1.196405	1.215901	1.584287	1.516092	2.145379	1.759987	1.474654	1.354109	1.609854	1.051915	1.164278	1.002558	1.603561	2.124461
PE 38:6	0.701031	0.627957	0.674041	0.884173	0.859776	1.109766	0.970030	0.770011	0.678929	0.833931	0.455421	0.486203	0.438127	0.713153	0.883036
PE 38:7	0.112398	0.093411	0.100726	0.139032	0.129146	0.160837	0.135796	0.102676	0.087565	0.127308	0.075778	0.083299	0.083101	0.121141	0.134117
PE 38:8	0.055149	0.052751	0.049147	0.075689	0.057787	0.072927	0.077832	0.058417	0.050226	0.057165	0.047573	0.047662	0.051118	0.067479	0.081312
PE 40:4	0.367748	0.403368	0.400120	0.534096	0.458819	0.509221	0.465343	0.452792	0.410387	0.484429	0.295120	0.336564	0.252404	0.425064	0.498021
PE 40:5	0.817726	0.914976	0.893042	1.213460	1.115525	1.193949	1.047184	1.007224	0.907469	1.040188	0.641044	0.715454	0.596962	0.897290	1.262927
PE 40:6	1.083035	1.302033	1.292169	1.682667	1.579853	1.719345	1.537117	1.286965	1.181109	1.391835	0.967470	1.011301	0.880133	1.215763	1.816885
PE 40:7	0.632423	0.745280	0.717770	1.074127	0.911284	1.059801	0.953299	0.714901	0.649992	0.805603	0.469377	0.504580	0.525932	0.625167	0.919010
PE 40:8	0.066204	0.076760	0.066867	0.125736	0.074688	0.084073	0.097668	0.075066	0.062490	0.072141	0.053919	0.052891	0.085606	0.069436	0.096110
SM 14:0	0.416466	0.549301	0.451527	0.586781	0.529508	0.554282	0.577234	0.397516	0.514479	0.534657	0.432622	0.448064	0.444496	0.325641	0.450544
SM 15:0	0.294139	0.360214	0.326794	0.399632	0.337563	0.363718	0.341296	0.257490	0.312015	0.346691	0.230544	0.253566	0.222805	0.203701	0.231272
SM 16:1	0.550698	0.766168	0.629036	0.722311	0.645682	0.718700	0.696121	0.528398	0.642048	0.667548	0.505200	0.543075	0.512483	0.426623	0.534033
SM 16:0	6.213556	8.157513	6.842692	8.444358	7.412087	7.650546	7.495955	5.702409	6.659156	7.173989	5.868219	6.420072	5.715151	4.734999	5.906423
dhSM 16:0	0.340721	0.402747	0.340973	0.366895	0.378662	0.485408	0.454871	0.360242	0.394519	0.598785	0.523104	0.546585	0.534806	0.377223	0.464767
SM 17:1	0.040418	0.045300	0.045648	0.052032	0.053632	0.043346	0.048034	0.037867	0.042126	0.042044	0.028046	0.031839	0.027419	0.024628	0.034196
SM 17:0	0.301628	0.410423	0.353989	0.415158	0.366462	0.375169	0.358179	0.297329	0.327635	0.355114	0.236759	0.262176	0.229262	0.207784	0.244390
dhSM 17:0	0.084834	0.121491	0.101734	0.120056	0.101502	0.101938	0.114111	0.103875	0.103574	0.114827	0.090139	0.087744	0.076318	0.068485	0.080823
SM 18:1	0.055284	0.087747	0.073801	0.084137	0.074156	0.075401	0.071787	0.049588	0.059242	0.065137	0.056015	0.056960	0.047108	0.046456	0.056390
SM 18:0	0.211052	0.273785	0.229024	0.273749	0.239198	0.258281	0.267680	0.190714	0.230783	0.219408	0.207199	0.214031	0.210690	0.178756	0.222099
SM 20:0	0.055438	0.087206	0.057365	0.063163	0.049379	0.057528	0.060111	0.045883	0.051511	0.051968	0.059098	0.067702	0.047735	0.038115	0.051963
SM 22:1	0.207116	0.258036	0.204138	0.273796	0.234555	0.238927	0.243210	0.177783	0.197959	0.212135	0.173238	0.206494	0.171018	0.151907	0.192235
SM 22:0	0.368979	0.587541	0.496677	0.636270	0.509255	0.449983	0.546192	0.404973	0.459512	0.399901	0.466061	0.481099	0.453157	0.407642	0.456476

Lipid Species	control #1	control #2	control #3	control #4	control #5	IL-1β #1	IL-1β #2	IL-1β #3	IL-1β #4	IL-1β #5	PDGF-BB #1	PDGF-BB #2	PDGF-BB #3	PDGF-BB #4	PDGF-BB #5
SM 23:1	0.210740	0.277302	0.222335	0.265038	0.237870	0.224409	0.227067	0.178226	0.207989	0.225317	0.161111	0.199900	0.166185	0.146967	0.164720
SM 23:0	0.116354	0.182110	0.138806	0.147065	0.155228	0.149659	0.160926	0.121299	0.128353	0.137414	0.111295	0.116976	0.094177	0.088545	0.116475
SM 24:2	0.628938	0.829081	0.695297	0.763906	0.710570	0.706709	0.706948	0.507408	0.580157	0.634645	0.508212	0.564066	0.474994	0.421425	0.515028
SM 24:1	2.387501	2.973725	2.503093	3.095227	2.669965	2.713020	2.717192	1.933647	2.227573	2.489223	2.108807	2.288717	1.962944	1.709238	2.044701
SM 24:0	0.602726	0.772818	0.708422	0.833158	0.731611	0.781932	0.746850	0.539888	0.604041	0.675713	0.673654	0.743927	0.628577	0.512212	0.647017
SM 25:1	0.087107	0.092308	0.092716	0.112407	0.106587	0.100107	0.113892	0.064689	0.076597	0.082117	0.079183	0.086737	0.071446	0.065037	0.088686
SM 25:0	0.034507	0.045172	0.037805	0.051766	0.034830	0.037491	0.043521	0.028074	0.030832	0.034784	0.032521	0.053095	0.033142	0.033558	0.037979
Cer 16:0	0.086968	0.103936	0.099318	0.115463	0.091580	0.109700	0.110974	0.081875	0.097427	0.096088	0.090824	0.085442	0.070393	0.074006	0.099349
Cer 18:0	0.003175	0.003692	0.003610	0.003865	0.003279	0.003153	0.003409	0.002269	0.002817	0.002771	0.002696	0.002925	0.002200	0.002395	0.003425
Cer 20:0	0.000809	0.001090	0.000943	0.001120	0.000961	0.001301	0.001236	0.000842	0.000996	0.001122	0.000797	0.000806	0.000598	0.000649	0.000805
Cer 22:0	0.008503	0.008368	0.008430	0.010675	0.008756	0.008379	0.008303	0.006250	0.007527	0.008625	0.008951	0.010597	0.008030	0.008307	0.009565
Cer 24:0	0.042851	0.044928	0.047080	0.063154	0.051643	0.053413	0.063923	0.041664	0.044967	0.052181	0.048819	0.051436	0.039115	0.038787	0.049541
Cer 24:1	0.055028	0.059865	0.063140	0.076642	0.070808	0.057675	0.057928	0.041287	0.045371	0.049352	0.039107	0.039318	0.031516	0.031536	0.044442
dhCer 16:0	0.005119	0.005700	0.006099	0.004874	0.004374	0.005724	0.006151	0.003795	0.004293	0.005751	0.003766	0.004468	0.003269	0.005555	0.005347
dhCer 18:0	0.000870	0.000795	0.000660	0.000479	0.000472	0.000380	0.000406	0.000203	0.000396	0.000473	0.000371	0.000466	0.000316	0.000549	0.000531
dhCer 22:0	0.000781	0.000920	0.000724	0.000753	0.000719	0.001046	0.000849	0.000654	0.000699	0.000614	0.000651	0.000930	0.000663	0.000857	0.000743
dhCer 24:0	0.001208	0.001506	0.001469	0.001521	0.001294	0.002025	0.002205	0.001497	0.001517	0.001874	0.001169	0.001476	0.001205	0.001152	0.001315
dhCer 24:1	0.001462	0.001904	0.001834	0.001847	0.001482	0.001625	0.001696	0.000926	0.001181	0.001382	0.000917	0.001079	0.000774	0.000836	0.001131
LacCer 16:0	0.016549	0.019477	0.020772	0.020896	0.018130	0.020026	0.019330	0.014551	0.018718	0.017718	0.012657	0.012867	0.011271	0.012015	0.014256
LacCer 18:0	0.001227	0.001488	0.001273	0.001573	0.001463	0.001601	0.001613	0.001160	0.001399	0.001344	0.001395	0.001385	0.001052	0.000917	0.001171
LacCer 20:0	0.000193	0.000293	0.000222	0.000131	0.000197	0.000212	0.000232	0.000131	0.000266	0.000206	0.000215	0.000160	0.000157	0.000139	0.000165
LacCer 22:0	0.018391	0.019897	0.023381	0.021515	0.018689	0.020879	0.022489	0.015882	0.022062	0.018108	0.014967	0.016600	0.015536	0.017394	0.016258
LacCer 24:0	0.025052	0.027941	0.027714	0.028784	0.025593	0.032092	0.031166	0.025515	0.025060	0.027445	0.020912	0.020444	0.019213	0.020978	0.030488
LacCer 24:1	0.055563	0.076734	0.071487	0.073721	0.055796	0.067812	0.058173	0.049439	0.062713	0.055049	0.041870	0.040087	0.035507	0.063356	0.058529
GluCer 16:0	0.064257	0.083581	0.081638	0.089945	0.084877	0.093157	0.095452	0.072264	0.079165	0.084685	0.048500	0.055111	0.048679	0.038859	0.047231
GluCer 18:0	0.004168	0.004971	0.004796	0.004876	0.004241	0.004652	0.004813	0.003321	0.003753	0.004241	0.003451	0.003613	0.003291	0.002734	0.003968
GluCer 20:0	0.000641	0.000702	0.000694	0.000922	0.000780	0.000846	0.000870	0.000604	0.000723	0.000846	0.000763	0.000854	0.000752	0.000627	0.000748
GluCer 22:0	0.024802	0.028652	0.029668	0.036461	0.030826	0.028312	0.031208	0.023130	0.024562	0.027629	0.023930	0.028749	0.024214	0.022722	0.025289
GluCer 24:0	0.037510	0.047717	0.049483	0.059494	0.052239	0.046492	0.049333	0.041327	0.047990	0.047209	0.038481	0.039485	0.038082	0.033039	0.040941
GluCer 24:1	0.060500	0.080598	0.073237	0.088526	0.073722	0.068272	0.070886	0.054673	0.058757	0.068444	0.038229	0.044306	0.041410	0.035771	0.037391
S1P	0.000652	0.000636	0.000574	0.000718	0.000728	0.000848	0.000768	0.000551	0.000496	0.000629	0.000711	0.000967	0.000892	0.000454	0.000532
dhS1P	0.000035	0.000066	0.000054	0.000057	0.000057	0.000068	0.000064	0.000036	0.000046	0.000053	0.000086	0.000117	0.000099	0.000067	0.000060
SG	0.137887	0.169685	0.155155	0.205572	0.190155	0.125400	0.127809	0.089829	0.098296	0.103005	0.074733	0.090823	0.067237	0.052638	0.068760
dhSG	0.011958	0.014094	0.011475	0.013802	0.012362	0.011541	0.009832	0.006466	0.006998	0.007540	0.006716	0.009048	0.006734	0.006904	0.007317
PhytoSG	0.001400	0.001532	0.001252	0.001845	0.001657	0.001874	0.001369	0.000906	0.000836	0.001139	0.000501	0.000663	0.000544	0.000420	0.000755

Supplementary Table S5. Data validation performed in silico using glomerular gene expression data from Nephroseq and glomerular mRNA sequencing data from Levin et al.

Gene	Pathway	Experimental Group	Species	FC	COPA	*p* value	*t* test
Acer2 (a)	Cer catabolism	db/db C57BLKS - DKD vs non-DKD	mouse	2.21		3.67E-04	6.12
Acer2 (c)	Cer catabolism	eNOS-deficient C57BLKS db/db - DKD vs non-DKD	mouse	1.98		3.12E-04	5.21
ASAH1 (e)	SM catabolism	DKD kidney vs healthy	human	0.51		5.46E-05	4.03
*CERK (25th) (d)	Cer phosphorylation	DKD kidney vs healthy	human		-1.29		
CERS6 (e)	Cer synthesis	DKD kidney vs healthy	human	-0.85		1.70E-04	-3.76
Degs2 (a)	SG catabolism	db/db C57BLKS - DKD vs non-DKD	mouse	-4.19		3.00E-03	-4.18
Degs2 (b)	SG catabolism	DBA/2 - DKD vs non-DKD	mouse	-1.82		1.13E-04	-5.73
Degs2 (c)	SG catabolism	eNOS-deficient C57BLKS db/db - DKD vs non-DKD	mouse	-3.22		1.00E-03	-4.56
*DGAT1 (5th) (d)	TG synthase	DKD kidney vs healthy	human		-4.49		
Elovl7 (a)	Cer synthesis	db/db C57BLKS - DKD vs non-DKD	mouse	-1.88		3.00E-03	-4.53
*Il1b (75th) (c)	Stimulant	eNOS-deficient C57BLKS db/db - DKD vs non-DKD	mouse		1.87		
*IL1B (90th) (d)	Stimulant	DKD kidney vs healthy	human		3.29		
Lpar1 (a)	LPA receptor	db/db C57BLKS - DKD vs non-DKD	mouse	1.79		2.78E-04	5.59
LPAR1 (e)	LPA receptor	DKD kidney vs healthy	human	1.43		1.24E-04	3.84
LPAR1 (f)	LPA receptor	DKD kidney vs healthy	human	1.91		8.57E-04	4.35
Lpar4 (a)	LPA receptor	db/db C57BLKS - DKD vs non-DKD	mouse	1.79		2.00E-03	4.70
LPAR6 (d)	LPA receptor	DKD kidney vs healthy	human	2.34		3.34E-08	8.45
*PDGFB (75th) (d)	Stimulant	DKD kidney vs healthy	human		1.20		
Pla2g4a (a)	PC catabolism	db/db C57BLKS - DKD vs non-DKD	mouse	2.11		2.00E-03	3.90
PLA2G4A (d)	PC catabolism	DKD kidney vs healthy	human	2.97		8.66E-06	5.68
PLA2G4A (f)	PC catabolism	DKD kidney vs healthy	human	1.86		5.00E-03	3.10
Ptger2 (c)	PG receptor	eNOS-deficient C57BLKS db/db - DKD vs non-DKD	mouse	-2.60		1.47E-04	-5.54
Ptger3 (c)	PG receptor	eNOS-deficient C57BLKS db/db - DKD vs non-DKD	mouse	1.89		6.47E-04	4.43
Ptger4 (c)	PG receptor	eNOS-deficient C57BLKS db/db - DKD vs non-DKD	mouse	-1.74		4.00E-03	-3.33
PTGER4 (d)	PG receptor	DKD kidney vs healthy	human	-1.53		6.00E-03	-3.00
PTGER4 (e)	PG receptor	DKD kidney vs healthy	human	-1.15		9.23E-07	-4.91
PTGER4 (f)	PG receptor	DKD kidney vs healthy	human	-3.79		3.17E-04	-5.11
Ptges (c)	PG synthesis	eNOS-deficient C57BLKS db/db - DKD vs non-DKD	mouse	1.93		9.00E-03	2.91
PTGES (f)	PG synthesis	DKD kidney vs healthy	human	1.82		8.03E-04	4.42
Ptgir (a)	PG receptor	db/db C57BLKS - DKD vs non-DKD	mouse	1.83		6.00E-03	3.33
PTGIR (e)	PG receptor	DKD kidney vs healthy	human	1.14		1.51E-05	4.33
Ptgis (a)	PG synthesis	db/db C57BLKS - DKD vs non-DKD	mouse	2.30		8.28E-05	6.66
Ptgis (c)	PG synthesis	eNOS-deficient C57BLKS db/db - DKD vs non-DKD	mouse	2.64		1.96E-05	7.09
*PTGIS (75th) (f)	PG synthesis	DKD kidney vs healthy	human		2.50		
Ptgs1/Cox1 (a)	PG synthesis	db/db C57BLKS - DKD vs non-DKD	mouse	1.97		9.71E-04	5.10
Ptgs1/Cox1 (c)	PG synthesis	eNOS-deficient C57BLKS db/db - DKD vs non-DKD	mouse	1.61		1.27E-05	6.44
*PTGS1/COX1 (75th) (f)	PG synthesis	DKD kidney vs healthy	human		2.64		
Ptgs2/Cox2 (a)	PG synthesis	db/db C57BLKS - DKD vs non-DKD	mouse	2.05		3.00E-03	4.72
Ptgs2/Cox2 (c)	PG synthesis	eNOS-deficient C57BLKS db/db - DKD vs non-DKD	mouse	1.91		4.50E-04	4.74
*PTGS2/COX2 (90th) (d)	PG synthesis	DKD kidney vs healthy	human		2.94		
SGPL1 (e)	PSB catabolism	DKD kidney vs healthy	human	0.50		3.88E-03	3.58
SGPL1 (f)	PSB catabolism	DKD kidney vs healthy	human	-1.66		5.00E-03	-3.03
Sgpp2 (c)	PSB catabolism	eNOS-deficient C57BLKS db/db - DKD vs non-DKD	mouse	1.58		9.00E-03	2.82

Gene	Pathway	Experimental Group	Species	FC	COPA	*p* value	*t* test
Sgms2 (a)	SM synthesis	db/db C57BLKS - DKD vs non-DKD	mouse	-1.59		1.40E-02	-3.22
SMPD2 (f)	SM catabolism	DKD kidney vs healthy	human	1.60		2.13E-05	5.89
SMPDL3B (b)	SM catabolism	DBA/2 - DKD vs non-DKD	mouse	1.74		5.95E-04	4.00
SMPDL3B (c)	SM catabolism	eNOS-deficient C57BLKS db/db - DKD vs non-DKD	mouse	2.79		2.13E-05	7.68
*SMPDL3B (90th) (d)	SM catabolism	DKD kidney vs healthy	human		2.78		
Smpdl3b (e)	SM catabolism	DKD kidney vs healthy	human	2.30		5.84E-03	3.44
Sphk1 (c)	SG phosphorylation	eNOS-deficient C57BLKS db/db - DKD vs non-DKD	mouse	2.16		4.94E-04	5.15
Sphk1 (d)	SG phosphorylation	DKD kidney vs healthy	human	1.62		2.08E-04	4.78
TBXA2R (e)	TX receptor	DKD kidney vs healthy	human	-0.90		4.20E-05	-4.10
TBXA2R (f)	TX receptor	DKD kidney vs healthy	human	-2.12		9.24E-04	-3.81
Tbxas1 (a)	TX synthesis	db/db C57BLKS - DKD vs non-DKD	mouse	-2.10		2.62E-06	-10.73

Human genes names are reported in uppercase, mouse genes names in lowercase. (a) Hodgin et al, [35] db/db C57BLKS mouse model - DKD vs non-DKD. (b) Hodgin et al, eNOS-deficient C57BLKS db/db mouse model - DKD vs non-DKD. (c) Hodgin et al, DBA/2 mouse model - DKD vs non-DKD. (d) Ju et al, [36] DKD human renal biopsies vs healthy individuals. (e) Levin et al, [29] DKD human renal biopsies vs healthy individuals. (f) Woroniecka et al, [37] DKD human renal biopsies vs healthy individuals. The asterisk before gene names indicates outliers' data. COPA values were plotted instead of FC in these cases.
